# Screen Anti-influenza Lead Compounds That Target the PA_C_ Subunit of H5N1 Viral RNA Polymerase

**DOI:** 10.1371/journal.pone.0035234

**Published:** 2012-08-24

**Authors:** Lin Li, Shenghai Chang, Junfeng Xiang, Qian Li, Huanhuan Liang, Yalin Tang, Yingfang Liu

**Affiliations:** 1 Beijing National Laboratory for Molecular Sciences (BNLMS), Center for Molecular Sciences, State Key Laboratory for Structural Chemistry of Unstable and Stable Species, Institute of Chemistry, Chinese Academy of Sciences, Beijing, China; 2 National Laboratory of Biomacromolecules, Institute of Biophysics, Chinese Academy of Sciences, Beijing, China; 3 Graduate University, Chinese Academy of Sciences, Beijing, P. R. China; Johns Hopkins School of Medicine, United States of America

## Abstract

The avian influenza (H5N1) viral RNA polymerase protein PA_C_ was used as a target to screen nine chlorogenic acid derivatives for their polymerase inhibitor activity. Among them, seven compounds were PA_C_ ligands, and four inhibited influenza RNA polymerase activity. These results aid in the design of anti-influenza agents based on caffeoylquinic acid.

## Introduction

Influenza virus, which has high rates of morbidity and mortality, is one of the major causes of viral respiratory infections. This virus mutates frequently, spreads rapidly, and occasionally transfers from animals to humans (e.g., avian influenza A, H5N1) [1]. The increasing geographic distribution of this epizootic virus has aroused serious concerns about the therapeutic methods currently available to curb a potential pandemic of this disease. To treat influenza, two classes of anti-influenza agents, M2 ion channel blockers and neuraminidase (NA) inhibitors, have been used [2], [3]. However, the emergence of drug-resistant viruses has limited the effectiveness of these drugs [4]–[6]. Therefore, alternative anti-influenza agents with a low risk of drug resistance are urgently needed.

In the search for a new generation of anti-influenza drugs, the RNA polymerase has been a valuable target to confront the disease [7]–[9]. This heterotrimer, which contains PB1, PB2 and PA subunits, is responsible for viral RNA (vRNA) replication and transcription. Structural analysis of the PA subunit revealed several potential active sites. Mutations of certain residues in these sites could significantly reduce the polymerase activity or disrupt vRNA replication [10]–[16]. Moreover, the structure of the PA subunit is conserved across type A, B and C influenza viruses. This conservation indicates that the anti-influenza agents targeting the PA subunit may be effective against most influenza strains and less susceptible to drug resistance [10]–[12]. Actually, some works have been done in the anti-influenza molecule screening targeting at PA [17]–[19]. In the pursuit of novel anti-influenza agents, we present a screen against the carboxyl-terminal domain of PA (termed PA_C_, residues 257–716).

## Results and Discussion

In previous work, we have discovered chlorogenic acid (CA) from *Flos Lonicera Japonica* and *Eucommia Ulmoides Oliv* extracts as a PA_C_ ligand and potential anti-influenza active compound [17]. Herein, nine CA derivatives, the principal active components of several anti-influenza traditional Chinese medicines (e.g., *Flos Lonicera Japonica* [20], *Stemona Japonica* [21]), were tested (Table 1, Table S1). To guide in the discovery of PA_C_ ligands, flexible docking simulations were utilized to evaluate the binding affinities between the compounds and the target. The docking was carried out using the AutoDock (v.4.01) software package with active sites established according to the structural analysis of PA_C_ [9]. The results were shown in Table S2 and Figure S9, S10, S11, S12, S13, S14, S15, S16, and S17, and binding affinities were summarized in Table 2. The binding affinities were scaled by p*K*_d_ values, where p*K*_d_ is the negative logarithm of the dissociation constant of the binding complex. Larger p*K*_d_ values indicate stronger binding affinities. The docking results suggested that candidate compounds **a**–**g** are potential binders of PA_C_.

**Table 1:** Candidate compounds in screening.

Candidates	Compound name
**a**	3,4-dicaffeoylquinic acid
**b**	1,5-dicaffeoylquinic acid
**c**	4,5-dicaffeoylquinic acid
**d**	3,5-dicaffeoylquinic acid
**e**	1,3-dicaffeoylquinic acid
**f**	5-caffeoylquinic acid
**g**	4-caffeoylquinic acid
**h**	quinic acid
**i**	caffeic acid

**Table 2:** Binding affinities of candidate compounds toward PA_C_ evaluated by visual docking, NMR, and SPR.

Cadidates	Visual docking (p*K*_d_)	NMR[a] (p*K*_d_)	SPR (p*K*_d_)
**a**	6.75	5.80	6.00
**b**	6.61	5.72	5.72
**c**	6.82	5.05	5.03
**d**	6.56	4.89	4.85
**e**	6.66	3.89	3.47
**f**	6.24	—	3.33
**g**	5.89	—	1.85
**h**	4.87	—	—
**i**	4.89	—	—

[a] Candidates **f** and **g** were identified to be PA_C_ ligands, but the binding affinities were too weak to be evaluated by NMR methods.

**Figure 1:**
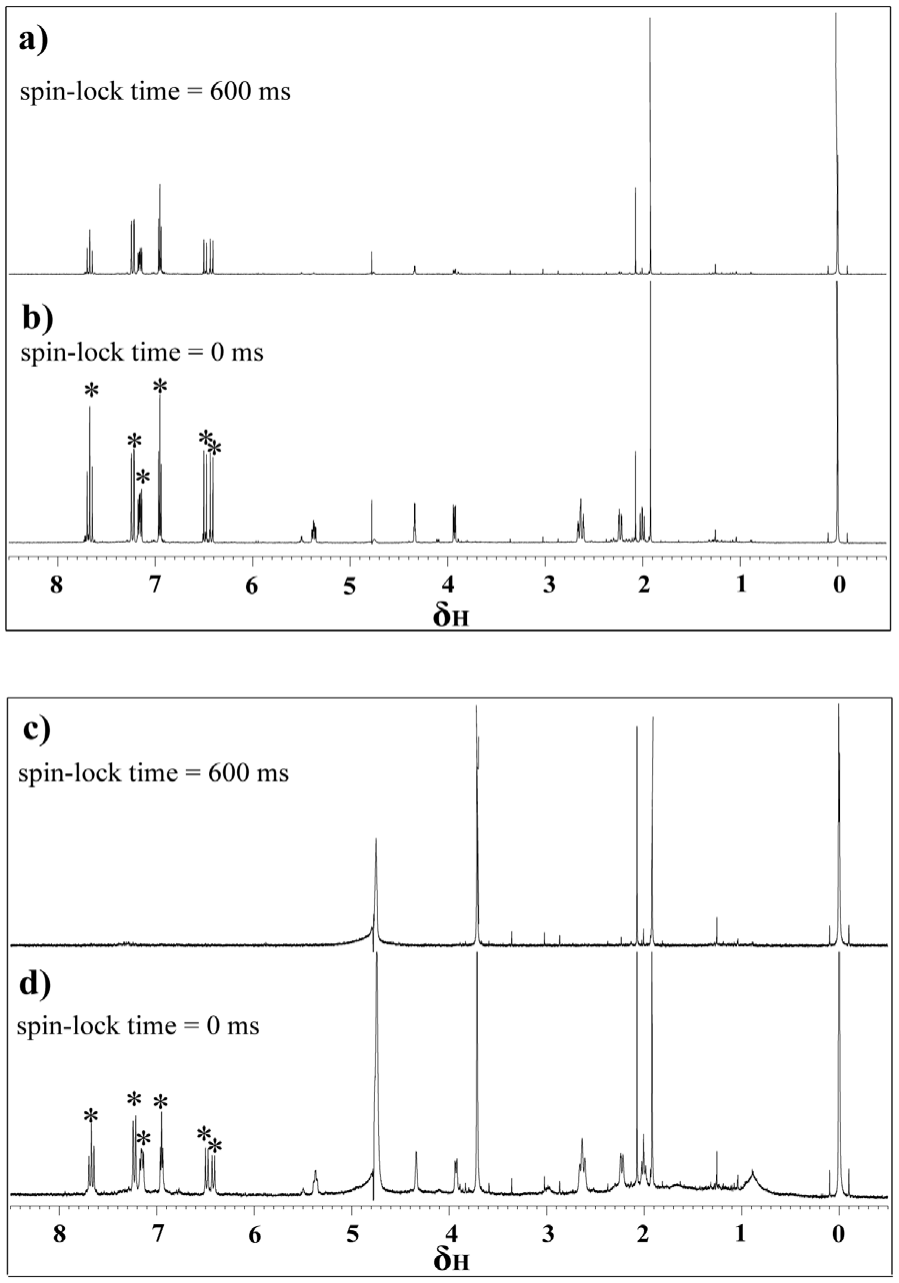
Binder screening by relaxation-edited NMR. Spectra of 1,5-dicaffeoylquinic acid (compound b) in the absence (plots **a**, **b**) and presence (plots **c**, **d**) of PA_C_, with the CPMG spin-lock time labeled beside each spectrum. The concentration of the small molecule and PA_C_ was 1.0×10^−3^ mol/L and 4.4×10^−6^ mol/L, respectively. The water peak located at δ 4.8 and 1 mM of TSP was added to the sample as a reference (δ 0). Signals of the small molecule in the absence of PA_C_ attenuated slightly when 600 ms of CPMG spin-lock was applied (plot **a**). These signals were eliminated at the same spin-lock time in the presence of PA_C_ (plot **c**). This difference should be ascribed to the increase in transverse relaxation rate (R_2_) of the small molecule upon its binding to PA_C_. The ligand peaks that attenuated when applying CPMG spin-lock in the presence of PA_C_ were marked with “*” in plots **b** and **d**.

The binding between the candidate compounds and PA_C_ was determined experimentally by analyzing the transverse relaxation change of the small molecule upon the addition of protein in a relaxation-edited NMR (Figure 1, Figure S1, S2, S3, S4, S5, S6, S7, and S8). As shown in Figure 1, the application of the Carr-Purcell-Meiboom-Gill (CPMG) spin-lock can suppress or even eliminate the fast-relaxing signals [22], [23] from the PA_C_ ligand. By using this method, seven of the nine compounds were confirmed to be PA_C_ ligands (candidates **a**, **b**, **c**, **d**, **e**, **f**, and **g**).

Afterward, the binding affinities of these compounds were evaluated by transverse relaxation simulation based on NMR (Figure S18, S19, S20, S21, and S22) and kinetics simulation in surface plasmon resonance (SPR) (Figure S23, S24, S25, S26, S27, S28, and S29). The results were summarized in Table 2. From this data, the disubstituted caffeoylquinic acids (**a**, **b**, **c**, **d**, **e**) exhibited higher binding affinities to PA_C_ relative to caffeic acid alone, quinic acid alone, and monosubstituted caffeoylquinic acids (**f**, **g**). Thus, the number and position of caffeoyls on quinic acid is an important factor in PA_C_ binding. On the other hand, although the docking simulation predicted that candidate **b** would bind tightly to the target, experimentally this compound behaved oppositely in affinity evaluation by NMR and SPR. This may be due to steric hindrance of the bulky trans-disubstitution of caffeoyl at the 1- and 3- position of the quinic acid. Other disubstitutedcaffeoyl quinic acids bind to PA_C_ with high affinities because the caffeoyls are either cis-disubstituted or both in equatorial positions.

In order to address specificity of the binding, the interaction between compound **a** and Human Serum Albumin (HSA) was evaluated by relaxation-edited NMR. As shown in Supporting Information (Figure S30), compound **a** did not specifically interact with HSA.

**Figure 2:**
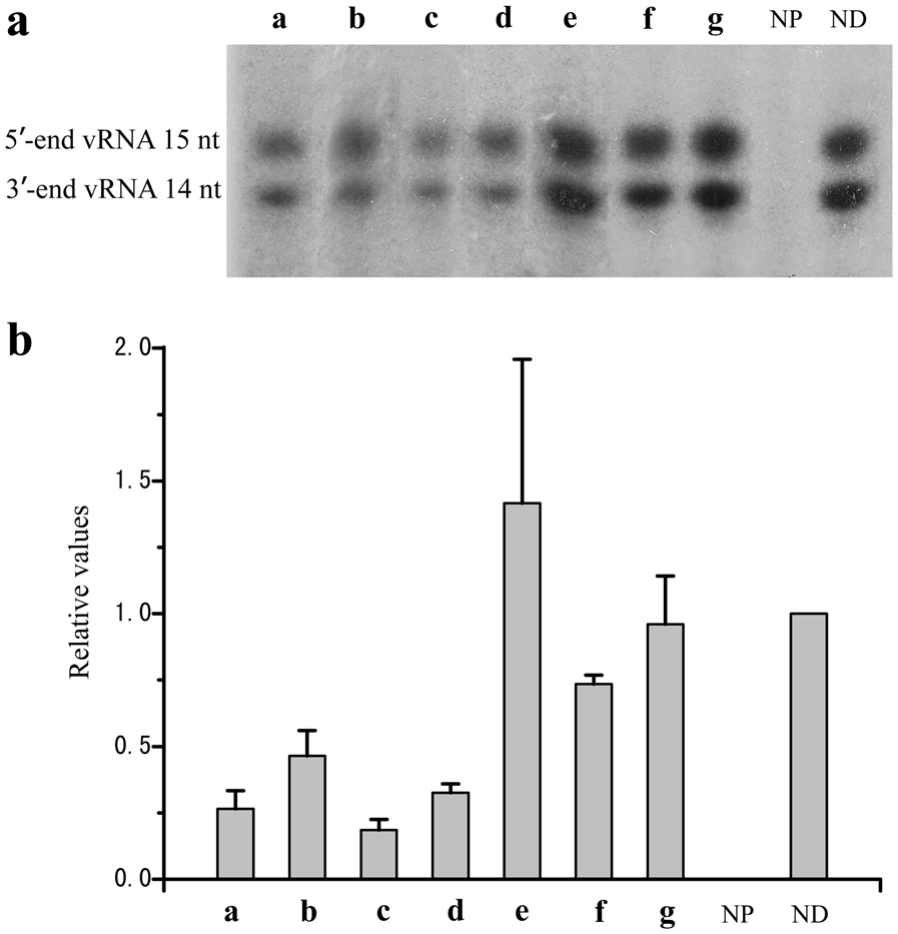
Results of ApG primer extension assay. (**a**) The effects of candidate compounds **a**, **b**, **c**, **d**, **e**, **f**, and **g** on polymerase activity in an ApG primer extension assay. The concentrations of the small molecules were 5 mM. NP stands for the negative reference with “no polymerase”, and ND for the positive reference with “no drug.” (**b**) The quantification of the results from (**a**) obtained by phosphorimaging analysis. The results are the average of two independent experiments and the derivations are shown.

The PA_C_ subunit is essential in influenza's RNA polymerase activity, so one may expect that PA_C_ ligands should inhibit RNA polymerase activity as well. The effects of these compounds on polymerase activity were evaluated by an ApG primer extension assay [24]. The polymerase can use ApG as a primer to synthesize cRNA from vRNA promoters; therefore, the length of cRNA can be used to judge polymerase activity. As shown in Figure 2, the candidate compounds **a**, **b**, **c**, and **d** inhibited the synthesis of cRNA while candidate **f** showed slight inhibition. The inhibition rates of compound **a**, **b**, **c**, **d**, and **f** on polymerase activity were 73%, 54%, 81%, 67%, and 26%, respectively. Notably, candidate **e** enhanced the polymerase activity at a rate of 42%. Although these compounds were assayed at relatively high concentrations (5 mM), the discovery of the inhibition effectiveness on polymerase activity of these CA derivatives suggests that CA could be a lead structure for potential anti-influenza drugs. These results clearly show that disubstituted caffeoylquinic acids with low steric hindrance are more likely to be effective inhibitors against polymerase because they bind strongly to PA_C_. It was also observed that some PA_C_ ligands did not exhibit activity in the ApG assay. This may well be due to their non-specific or weak binding to PA_C_.

In summary, the avian influenza viral RNA polymerase protein PA_C_, a conserved key target in the design of a new generation of anti-influenza agents, was used to screen lead plant-derived anti-influenza compounds. Seven compounds were identified as PA_C_ ligands. Among them, four compounds inhibited polymerase activity. Therefore, PA_C_ made a useful target to screen for anti-influenza agents. Based on the structure-activity relationship of CA derivatives as polymerase inhibitors, the position and number, and maybe also the synergistic effect, of the caffeoyls in quinic acid played important roles in the inhibition potential of polymerase activity. These results provided an important step in caffeoylquinic acid structure-based design of anti-influenza agents.

## Materials and Methods

### Materials

1,5-dicaffeoylquinic acid, 1,3-dicaffeoylquinic acid, 3,5-dicaffeoylquinic acid, 3,4-dicaffeoylquinic acid, 4,5-dicaffeoylquinic acid, 5-caffeoylquinic acid, 4-caffeoylquinic acid, quinic acid, and caffeic acid were purchased from Chengdu Mansite Pharmacetical Co., Ltd. (Sichuan, China). D_2_O and 3-(trimethylsilyl) propionic acid-d_4_ (TSP-d_4_) sodium salt were purchased from Sigma Co. (USA).

### Protein expression and purification

Methods for the preparation of PA_C_ protein were previously described [9]. Briefly, residues 257–716 of the PA subunit of avian H5N1 influenza A virus (A/goose/Guangdong/1/96) were cloned into a pGEX-6p vector (GE Healthcare) and transformed into *Escherichia coli* strain BL21. Cells were cultured in LB medium at 37°C with 100 mg/L of Ampicillin. When the OD600 reached 0.6–0.8, the culture was induced with 0.5 mM isopropyl-thio-D-glactosidase (IPTG) at 16°C. After 20 hours of incubation, the cells expressing PA_C_ were harvested and combined by centrifugation at 5000 rpm for 10 min. Recombinant protein was purified with a glutathione affinity column (GE Healthcare). Glutathione S-transferase (GST) was cleaved with PreScission protease (GE Healthcare), and the protein complex was further purified by Q sepharose FF ion exchange chromatography and Superdex-200 gel filtration chromatography (GE Healthcare). Methods for the preparation of the RNA polymerase were previously described [25]. Briefly, the RNA polymerase (PA, PB1, and PB2) complex was expressed in hi5 insect cell and purified by Ni-affinity column, ion-exchange column, and gel exclusion chromatography.

### The affinity analysis based on virtual docking

To predict the binding affinities between candidate compounds and PA_C_, the simulated flexible docking of ligands was carried out using the AutoDock (v.4.01) software package. The structure of PA_C_ was retrieved from the Protein Data Bank (PDB entries: 3CM8) and modified for visual docking. First, in the PDB file, water molecules were removed, polar hydrogen atoms were added, and non-polar hydrogen atoms were merged using the Hydrogen module in the AutoDock Tools (ADT). Then, Kollman united atom partial charges were assigned. The grid map of the docking simulation was established in a 61×61×61 cube centered on the target active sites referred to in a previous report [10]. The targets are defined as site 1: center of K328, K539, R566 and K574; site 2: center of K539, R566, K574 and N696; site 3: center of E410, K461, E524 and K536; site 4: center of F411, M595, L666, W706, F710, V636 and L640; and site 5: center of 620 and 621. There is a spacing of 0.375 Å between the grid points. When the ligand was docked to the PA_C_ target, the Lamarckian genetic algorithm was used to optimize the conformation of the ligand in the binding pocket. The parameters were set to the following: the size of the population was 150; the number of energy evaluations was set to 1.0×10^8^ as the run terminates; for clustering the conformations, the root mean square deviation tolerance was set to 2.0; fifty independent docking runs were carried out for every ligand; and other parameters were set to default. The binding affinities of the candidate compounds to the targets were summarized in Table S2, and the average p*K*_i_ of the five active sites was summarized in Table 2.

### NMR experiments

NMR experiments were performed on a Bruker AVANCE 600 spectrometer equipped with a 5 mm BBI probe capable of delivering z-field gradients, using TOPSPIN software (Bruker, version 2.1) was used in experimental manipulating and data processing. All experiments were carried out at 298.2 K. The relaxation-edited NMR experiments utilized a [*D*/pre-saturation-90*_x_*-(Δ-180*_y_*-Δ)*_n_*-acquire] pulse sequence, in which the CPMG sequence was used for the spin-lock. For all relaxation-edited experiments the following variables were used: pre-saturation water suppression was applied in pre-acquisition delay (*D* = 3 s), *P*_90_ was measured and set up for each sample, Δ = 1.5 ms, and 2×*n*×Δ = total spin-lock time. The spectra were collected with 64 k of data points and 32 scans. Transverse relaxation times were measured by a pseudo-2D experiment using the CPMG sequence. Presaturation was applied in a pre-acquisition delay for water suppression, and 32 k and 16 data points were set for the F2 and F1 dimension, respectively. In each experiment, the following variables were used: *D* = 3 s (pre-acquisition delay); *P*_90_ was measured and set up for each sample; Δ = 1.5 ms; the list of spin-lock loop (*n*) was set to 0, 10, 20, 30, 40, 50, 80, 100, 150, 200, 250, 300, 350, 400, 500, 600; and 2×*n*×Δ = total spin-lock time. The spectra were collected with 32 scans, and each *T*_2_ was calculated using TOPSPIN (Bruker, version 2.1) software by simulating the peak attenuation curve in different spin-lock times.

### The affinity analysis based on *T*_2_ simulation

The method to evaluate *K*_d_ by *T*_2_ simulation was previously reported [26]. When a ligand (L) and a protein (P) form a complex (LP), there is a dissociation equilibrium LP = L+P in the solution. The dissociation constant, *K*_d_, of this equilibrium is a key factor to describe the binding strength of the ligand and protein. It can be evaluated by the transverse relaxation time (*T*_2_) of the ligand in the presence of the protein. The expression describing the observed effective transverse relaxation rate *R*_2obs_ (1/*T*_2obs_) as a function of molar ratio of protein to ligand (*C*_P_/*C*_L_) is:
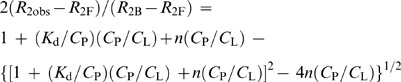



Since the total concentration of PA_C_, *C*_P_, and the ligand, *C*_L_, in the experiments were known, it is possible to obtain the *T*_2F_ and *T*_2B_ from the plot of *T*_2obs_ versus *C*_P_/*C*_L_. Extrapolation of the curve to *C*_P_/*C*_L_ = 0 should give *T*_2F_ and to infinite give *T*_2B_. Then, by simulating the plot of *R*_2obs_ versus *C*_P_/*C*_L_ using the above equation, *K*_d_ can be simultaneously obtained.

To evaluate the binding affinities between the candidate compounds and PA_C_, a series of samples containing small molecules and PA_C_ with different concentration ratios (shown in Figure S9, S10, S11, S12, and S13) were prepared. These *T*_2_ of the ligands were determined by the CPMG method using NMR. As we can see from Figure S9, S10, S11, S12, and S13, the observed *T*_2_ (*T*_2obs_) of the ligand decreases when the concentration ratio of protein to ligand (*C*_P_/*C*_L_) increases and the variation trends according to exponential decay. By simulating the plot of *R*_2obs_ (*R*_2obs_ = 1/*T*_2obs_) versus *C*_P_/*C*_L_ using equation (6), the *K*_d_ value can be obtained. The results of the simulation were summarized in Table 2.

### The affinity analysis based on Surface Plasmon Resonance (SPR)

An affinity analysis of the interaction of candidate compounds a-g and PA_C_ was carried out using an SPR spectrometer (Biacore 3000, GE Healthcare Bio-Sciences, Sweden). PA_C_ at a concentration of 10 µg/mL in 10 mM NaAC, pH 5.5, was used to couple this protein to a CM5 sensor chip. To determine the affinity of small molecules to PA_C_, increasing concentrations (labeled in Figure S14, S15, S16, S17, S18, S19, and S20) of the small molecules diluted in running buffer (130 mM PBS, pH 7.5) were injected over the sensor chip for 60 s (association phase), which was followed by dissociation for 180 s and recording of the spectra. All of the experiments were performed at 25°C, and the flow rate was 30 µL/min. The surface at the end of each experiment was regenerated using 20 mM NaOH at 30 µL/min for 10 s to remove any bound analyte. The data were analyzed using the BIAevaluation software (4.1 version) to calculate the affinity constant. The association and dissociation kinetics plots of the small molecules to PA_C_ were displayed in Figure S14, S15, S16, S17, S18, S19, and S20, and the results were summarized in Table 2.

### Polymerase activity test

The ApG primer extension assay was previously described [24]. We performed 5 µL reactions with 2.5 µL 3P and 0.7 µM model vRNA promoter (an equimolar mixture of the 5′-end vRNA 5′-AGUAGAAACAAGGCC-3′ and 3′-end vRNA 5′-GGCCUGCUUUUGCU-3′) in the presence of 5 mM MgCl_2_, 5 mM drug, 5 mM dithiothreitol, 1 mM ATP, 0.5 mM UTP, 0.5 mM CTP, 0.1 µM[α-^32^P]GTP (3,000 Ci/mmol), and 2 U/µL RNasin (Promega). Where indicated, 0.5 mM ApG (Sigma) was added to the reaction. The reaction system can be described as: 0.25 µL 0.1 M MgCl_2_, 0.25 µL 0.1 M DTT, 0.5 µL 10 mM ApG, 0.25 µL RNasin, 0.25 µL 20×NTP, 0.25 µL 20×promoter, 0.25 µL [α-^32^P]GTP, 2.5 µL polymerase, 0.5 µL depc H_2_O or 0.5 µL small molecule. Reactions were incubated at 30°C for 1 h. The loading buffer, 5 µL 2×formamide/bromophenolblue/EDTA, and the mixture were heated at 95°C for 2 min. Analysis was performed by running the samples on an 18% PAGE with 1×Tris-borate-EDTA and 8 M urea followed by autoradiography.

## Supporting Information

Figure S1 **Binder screening by relaxation-edited NMR.** Spectra of 3,4-dicaffeoylquinic acid (compound **a**) in the absence (plots ***a*** *,* ***b***) and presence (plots ***c*** *,* ***d***) of PA_C_. The CPMG spin-lock time of each experiment was labeled beside the spectrum. The concentration of the small molecule and PA_C_ was 1.0×10^−3^ mol/L and 7.0×10^−6^ mol/L, respectively. The water peak located at δ 4.8 and 1 mM of TSP was added to the sample as a reference (δ 0). The ligand peaks that attenuated when applying CPMG spin-lock in the presence of PA_C_ were marked with “*” in plot ***d***.
(TIF)

Figure S2 **Binder screening by relaxation-edited NMR.** Spectra of 4,5-dicaffeoylquinic acid (compound **c**) in the absence (plots ***a*** *,* ***b***) and presence (plots ***c*** *,* ***d***) of PA_C_. The CPMG spin-lock time of each experiment was labeled beside the spectrum. The concentration of the small molecule and PA_C_ was 1.0×10^−3^ mol/L and 7.0×10^−6^ mol/L, respectively. The water peak located at δ 4.8 and 1 mM of TSP was added to the sample as a reference (δ 0). The ligand peaks that attenuated when applying CPMG spin-lock in the presence of PA_C_ were marked with “*” in plot ***d***.
(TIF)

Figure S3 **Binder screening by relaxation-edited NMR.** Spectra of 3,5-dicaffeoylquinic acid (compound **d**) in the absence (plots ***a*** *,* ***b***) and presence (plots ***c*** *,* ***d***) of PA_C_. The CPMG spin-lock time of each experiment was labeled beside the spectrum. The concentration of the small molecule and PA_C_ was 1.0×10^−3^ mol/L and 7.0×10^−6^ mol/L, respectively. The water peak located at δ 4.8 and 1 mM of TSP was added to the sample as a reference (δ 0). The ligand peaks that attenuated when applying CPMG spin-lock in the presence of PA_C_ were marked with “*” in plot ***d***.
(TIF)

Figure S4 **Binder screening by relaxation-edited NMR.** Spectra of 1,3-dicaffeoylquinic acid (compound **e**) in the absence (plots ***a*** *,* ***b***) and presence (plots ***c*** *,* ***d***) of PA_C_. The CPMG spin-lock time of each experiment was labeled beside the spectrum. The concentration of the small molecule and PA_C_ was 1.0×10^−3^ mol/L and 5.5×10^−6^ mol/L, respectively. The water peak located at δ 4.8 and 1 mM of TSP was added to the sample as a reference (δ 0). The ligand peaks that attenuated when applying CPMG spin-lock in the presence of PA_C_ were marked with “*” in plots ***d***.
(TIF)

Figure S5 **Binder screening by relaxation-edited NMR.** Spectra of 5-caffeoylquinic acid (compound **f**) in the absence (plots ***a*** *,* ***b***) and presence (plots ***c*** *,* ***d***) of PA_C_. The CPMG spin-lock time of each experiment was labeled beside the spectrum. The concentration of the small molecule and PA_C_ was 1.0×10^−3^ mol/L and 8.7×10^−6^ mol/L, respectively. The water peak located at δ 4.8 and 1 mM of TSP was added to the sample as a reference (δ 0). The ligand peaks that attenuated when applying CPMG spin-lock in the presence of PA_C_ were marked with “*” in plot ***d***.
(TIF)

Figure S6 **Binder screening by relaxation-edited NMR.** Spectra of 4-caffeoylquinic acid (compound **g**) in the absence (plots ***a*** *,* ***b***) and presence (plots ***c*** *,* ***d***) of PA_C_. The CPMG spin-lock time of each experiment was labeled beside the spectrum. The concentration of the small molecule and PA_C_ was 1.0×10^−3^ mol/L and 8.7×10^−6^ mol/L, respectively. The water peak located at δ 4.8 and 1 mM of TSP was added to the sample as a reference (δ 0). The ligand peaks that attenuated when applying CPMG spin-lock in the presence of PA_C_ were marked with “*” in plot ***d***.
(TIF)

Figure S7 **Binder screening by relaxation-edited NMR.** Spectra of quinic acid (compound **h**) in the absence (plots ***a*** *,* ***b***) and presence (plots ***c*** *,* ***d***) of PA_C_. The CPMG spin-lock time of each experiment was labeled beside the spectrum. The concentration of the small molecule and PA_C_ was 1.0×10^−3^ mol/L and 8.7×10^−6^ mol/L, respectively. The water peak located at δ 4.8 and 1 mM of TSP was added to the sample as a reference (δ 0).
(TIF)

Figure S8 **Binder screening by relaxation-edited NMR.** Spectra of caffeic acid (compound **i**) in the absence (plots ***a*** *,* ***b***) and presence (plots ***c*** *,* ***d***) of PA_C_. The CPMG spin-lock time of each experiment was labeled beside the spectrum. The concentration of the small molecule and PA_C_ was 1.0×10^−3^ mol/L and 8.7×10^−6^ mol/L, respectively. The water peak located at δ 4.8 and 1 mM of TSP was added to the sample as a reference (δ 0).
(TIF)

Figure S9 **The most possible binding site of 3,4-dicaffeoylquinic acid on PA_C_ supposed by visual docking.**
(TIF)

Figure S10 **The most possible binding site of 1,5-dicaffeoylquinic acid on PA_C_ supposed by visual docking.**
(TIF)

Figure S11 **The most possible binding site of 4,5-dicaffeoylquinic acid on PA_C_ supposed by visual docking.**
(TIF)

Figure S12 **The most possible binding site of 3,5-dicaffeoylquinic acid on PA_C_ supposed by visual docking.**
(TIF)

Figure S13 **The most possible binding site of 1,3-dicaffeoylquinic acid on PA_C_ supposed by visual docking.**
(TIF)

Figure S14 **The most possible binding site of 5-caffeoylquinic acid on PA_C_ supposed by visual docking.**
(TIF)

Figure S15 **The most possible binding site of 4-caffeoylquinic acid on PA_C_ supposed by visual docking.**
(TIF)

Figure S16 **The most possible binding site of quinic acid on PA_C_ supposed by visual docking.**
(TIF)

Figure S17 **The most possible binding site of caffeic acid on PA_C_ supposed by visual docking.**
(TIF)

Figure S18 **Binding affinity between 1,5-dicaffeoylquinic acid and PA_C_ evaluated by transverse relaxation simulation.** **a**) The plot of *T*_2obs_ versus *C*_P_/*C*_L_; **b**) The plot of *R*_2obs_ versus *C*_P_/*C*_L_.
(TIF)

Figure S19 **Binding affinity between 1,3-dicaffeoylquinic acid and PA_C_ evaluated by transverse relaxation simulation.** **a**) The plot of *T*_2obs_ versus *C*_P_/*C*_L_; **b**) The plot of *R*_2obs_ versus *C*_P_/*C*_L_.
(TIF)

Figure S20 **Binding affinity between 3,5-dicaffeoylquinic acid and PA_C_ evaluated by transverse relaxation simulation.** **a**) The plot of *T*_2obs_ versus *C*_P_/*C*_L_; **b**) The plot of *R*_2obs_ versus *C*_P_/*C*_L_.
(TIF)

Figure S21 **Binding affinity between 3,4-dicaffeoylquinic acid and PA_C_ evaluated by transverse relaxation simulation.** **a**) The plot of *T*_2obs_ versus *C*_P_/*C*_L_; **b**) The plot of *R*_2obs_ versus *C*_P_/*C*_L_.
(TIF)

Figure S22 **Binding affinity between 4,5-dicaffeoylquinic acid and PA_C_ evaluated by transverse relaxation simulation.** **a**) The plot of *T*_2obs_ versus *C*_P_/*C*_L_; **b**) The plot of *R*_2obs_ versus *C*_P_/*C*_L_.
(TIF)

Figure S23 **Association and dissociation kinetics plot of 3,4-dicaffeoylquinic acid to PAC as determined by SPR.**
(TIF)

Figure S24 **Association and dissociation kinetics plot of 1,5-dicaffeoylquinic acid to PAC as determined by SPR.**
(TIF)

Figure S25 **Association and dissociation kinetics plot of 4,5-dicaffeoylquinic acid to PAC as determined by SPR.**
(TIF)

Figure S26 **Association and dissociation kinetics plot of 3,5-dicaffeoylquinic acid to PAC as determined by SPR.**
(TIF)

Figure S27 **Association and dissociation kinetics plot of 1,3-dicaffeoylquinic acid to PAC as determined by SPR.**
(TIF)

Figure S28 **Association and dissociation kinetics plot of 5-caffeoylquinic acid to PA_C_ as determined by SPR.**
(TIF)

Figure S29 **Association and dissociation kinetics plot of 4-caffeoylquinic acid to PA_C_ as determined by SPR.**
(TIF)

Figure S30 **Binder screening by relaxation-edited NMR.** Spectra of 3,4-dicaffeoylquinic acid (compound **a**) in the absence (plots ***a*** *,* ***b***) and presence (plots ***c*** *,* ***d***) of HSA. The CPMG spin-lock time of each experiment was labeled beside the spectra. The concentrations of small molecule and HSA were 1.0×10^−3^ mol/L and 7.0×10^−6^ mol/L, respectively. The water peak located at δ 4.8. The peaks of small molecule were not eliminated at a long spin-lock time, indicated that compound **a** did not specifically interact with HSA.
(TIF)

Table S1 **Structure of candidate compounds.**
(DOC)

Table S2 **Binding affinities of chlorogenic acid to different active sites of PA_C_ evaluated by virtual docking.**
(DOC)

## References

[1] Subbarao K, Klimov A, Katz J, Regnery H, Lim W, et al (1998) Characterization of an avian influenza A (H5N1) virus isolated from a child with a fatal respiratory illness. Science 279: 393–396. 9430591 10.1126/science.279.5349.393

[2] De Clercq E (2006) Antiviral agents active against influenza A viruses. Nat Rev Drug Discov 5: 1015–1025. 17139286 10.1038/nrd2175 PMC7097821

[3] De Clercq E, Neyts J (2007) Avian influenza A (H5N1) infection: targets and strategies for chemotherapeutic intervention. Trends Pharmacol Sci 28: 280–285. 17481739 10.1016/j.tips.2007.04.005 PMC7112898

[4] Kiso M, Mitamura K, Sakai-Tagawa Y, Shiraishi K, Kawakami C, et al (2004) Resistant influenza A viruses in children treated with oseltamivir: descriptive study. Lancet 364: 759–765. 15337401 10.1016/S0140-6736(04)16934-1

[5] Le QM, Kiso M, Someya K, Sakai YT, Nguyen TH, et al (2005) Avian flu: isolation of drug-resistant H5N1 virus. Nature 437: 1108. 16228009 10.1038/4371108a

[6] De Jong MD, Thanh TT, Khanh TH, Hien VM, Smith GJD, et al (2005) Brief report: Oseltamivir resistance during treatment of influenza A (H5N1) infection. New Engl J Med 353: 2667–2672. 16371632 10.1056/NEJMoa054512

[7] Obayashi E, Yoshida H, Kawai F, Shibayama N, Kawaguchi A, et al (2008) The structure basis for an essential subunit interaction in influenza virus RNA polymerase. Nature 454: 1127–1132. 18660801 10.1038/nature07225

[8] Dias A, Bouvier D, Crépin T, McCarthy AA, Hart DJ, et al (2009) The cap-snatching endonuclease of influenza virus polymerase resides in the PA subunit. Nature 458: 914–918. 19194459 10.1038/nature07745

[9] He XJ, Zhou J, Bartlam M, Zhang RG, Ma JY, et al (2008) Crystal structure of the polymerase PAC–PB1N complex from an avian influenza H5N1 virus. Nature 454: 1123–1127. 18615018 10.1038/nature07120

[10] Yuan PW, Bartlam M, Lou ZY, Chen SD, Zhou J, et al (2009) Crystal structure of an avian influenza polymerase PAN reveals an endonuclease active site. Nature 458: 909–914. 19194458 10.1038/nature07720

[11] Nakazawa M, Kadowaki SE, Watanabe I, Kadowaki Y, Takei M, et al (2008) PA subunit of RNA polymerase as a promising target for anti-influenza virus agents. Antivir Res 78: 194–201. 18258312 10.1016/j.antiviral.2007.12.010

[12] Fodor E, Crow M, Mingay LJ, Deng T, Sharps J, et al (2002) A single amino acid mutation in the PA subunit of the influenza virus RNA polymerase inhibits endonucleolytic cleavage of capped RNAs. J Virol 76: 8989–9001. 12186883 10.1128/JVI.76.18.8989-9001.2002 PMC136441

[13] Hara K, Schmidt FI, Crow M, Brownlee GG (2006) Amino acid residues in the N-terminal region of the PA subunit of influenza A virus RNA polymerase play a critical role in protein stability, endonuclease activity, cap binding, and virion RNA promoter binding. J Virol 80: 7789–7798. 16873236 10.1128/JVI.00600-06 PMC1563815

[14] Hara K, Shiota M, Kido H, Ohtsu Y, Kashiwagi T, et al (2001) Influenza virus RNA polymerase PA subunit is a novel serine protease with Ser624 at the active site. Genes Cells 6: 87–97. 11260254 10.1046/j.1365-2443.2001.00399.x

[15] Rodriguez A, Perez-Gonzalez A, Nieto A (2007) Influenza Virus Infection Causes Specific Degradation of the Largest Subunit of Cellular RNA Polymerase II. J Virol 81: 5315–5324. 17344288 10.1128/JVI.02129-06 PMC1900203

[16] Regan JF, Liang Y, Parslow TG (2006) Defective assembly of Influenza A virus due to a mutation in the polymerase subunit PA. J Virol 80: 252–261. 16352550 10.1128/JVI.80.1.252-261.2006 PMC1317532

[17] Li L, Chang SH, Xiang JF, Li Q, Liang HH, et al (2011) Screening Anti-influenza Agents that Target at Avian Influenza Polymerase Protein PA_C_ from Plant Extracts Based on NMR Methods. Chem Lett 40: 801–803.

[18] Iwai Y, Murakami K, Gomi Y, Hashimoto T, Asakawa Y, et al (2011) Anti-Influenza Activity of Marchantins, Macrocyclic Bisbibenzyls Contained in Liverworts. PLoS ONE 6 5:e19825. 21625478 10.1371/journal.pone.0019825 PMC3098833

[19] Su CY, Cheng RTJ, Lin MI, Wang SY, Huang WI, et al (2010) High-throughput Identification of Compounds Targeting Influenza RNA-Dependent RNA Polymerase Activity. PNAS 107: 19151–19156. 20974907 10.1073/pnas.1013592107 PMC2984200

[20] Ko HC, Wei BL, Chiou WF (2006) The effect of medicinal plants used in Chinese folk medicine on RANTES secretion by virus-infected human epithelial cells. J Ethnopharmacol 107: 205–210. 16621378 10.1016/j.jep.2006.03.004

[21] Ge F, Ke CQ, Tang W, Yang XZ, Tang CP, et al (2007) Isolation of chlorogenic acids and their derivatives from stemona japonica by preparative HPLC and evaluation of their anti-AIV (H5N1) activity in vitro. Phytochem Anal 18: 213–218. 17500364 10.1002/pca.974

[22] Lepre CA, Moore JM, Peng JW (2004) Theory and applications of NMR-based screening in pharmaceutical research. Chem Rev 104: 3641–3675. 15303832 10.1021/cr030409h

[23] Meyer B, Peters T (2003) NMR spectroscopy techniques for screening and identifying ligand binding to protein receptors. Angew Chem Int Ed 42: 864–890. 10.1002/anie.200390233 12596167

[24] Deng T, Vreede FT, Brownlee GG (2006) Different de novo initiation strategies are used by influenza virus RNA polymerase on its cRNA and viral RNA promoters during viral RNA replication. J Virol 80: 2337–2348. 16474140 10.1128/JVI.80.5.2337-2348.2006 PMC1395412

[25] Zhang SJ, Wang JL, Wang Q, Toyoda T (2010) Internal initiation of influenza virus replication of viral RNA and complementary RNA *in vitro* . J Bio Chem 285: 41194–41201. 20858902 10.1074/jbc.M110.130062 PMC3003417

[26] Luo RS, Liu ML, Mao XA (1999) NMR diffusion and relaxation study of drug-protein interaction. Spectrochim Acta A 55: 1897–1901. 10.1016/s1386-1425(99)00052-9 10474906

