# Supplementary figures and images for: Screen Anti-influenza Lead Compounds That Target the PAC Subunit of H5N1 Viral RNA Polymerase
Source: PLoS One. 2012 Aug 24;7(8):e35234. doi: 10.1371/journal.pone.0035234 (PMC3427309; doi:10.1371/journal.pone.0035234)

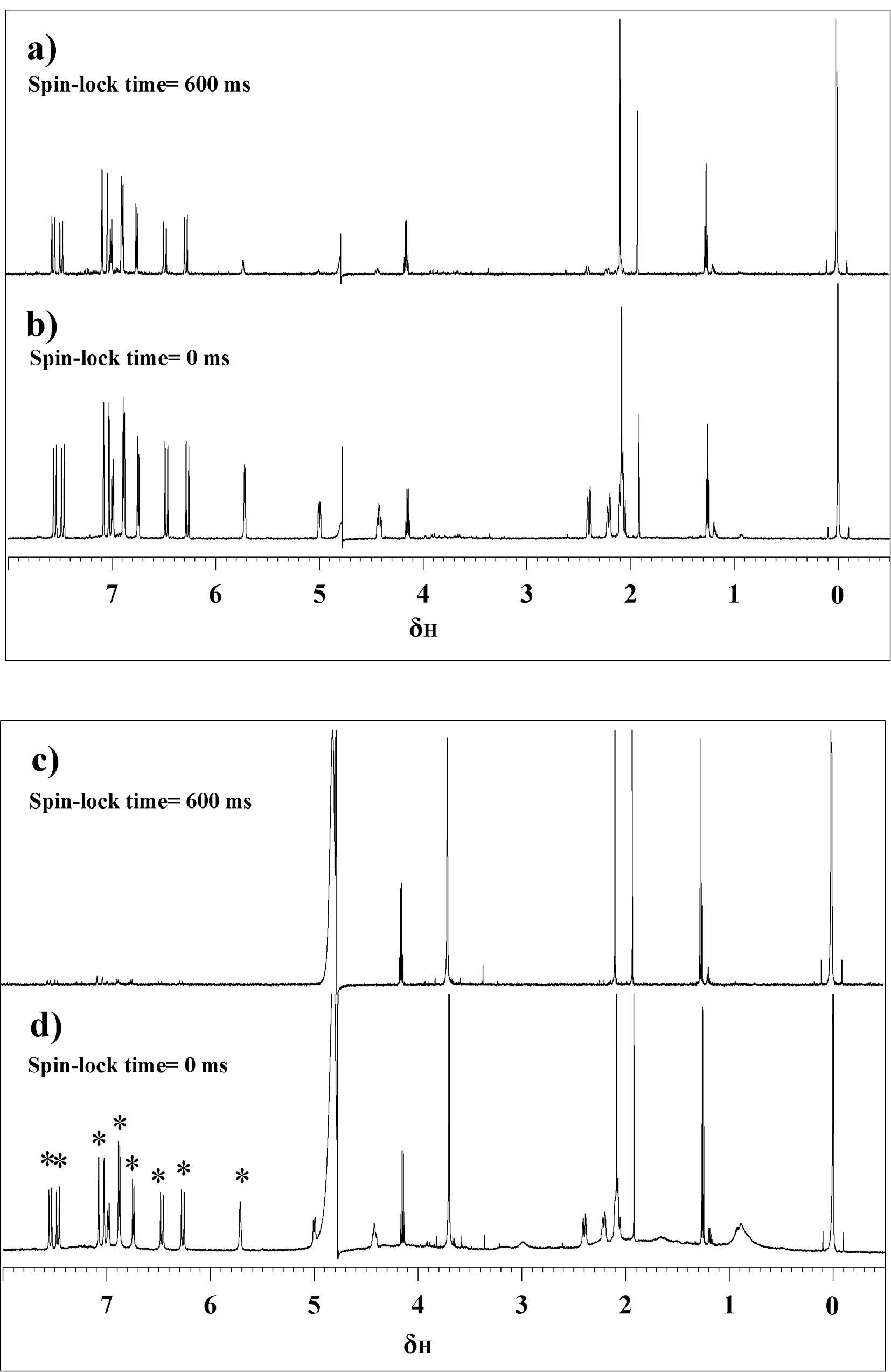

Supplement: Figure S1 — Binder screening by relaxation-edited NMR. Spectra of 3,4-dicaffeoylquinic acid (compound a) in the absence (plots a , b) and presence (plots c , d) of PAC. The CPMG spin-lock time of each experiment was labeled beside the spectrum. The concentration of the small molecule and PAC was 1.0×10^−3^ mol/L and 7.0×10^−6^ mol/L, respectively. The water peak located at δ 4.8 and 1 mM of TSP was added to the sample as a reference (δ 0). The ligand peaks that attenuated when applying CPMG spin-lock in the presence of PAC were marked with “*” in plot d. (TIF) [file pone.0035234.s001.tif]

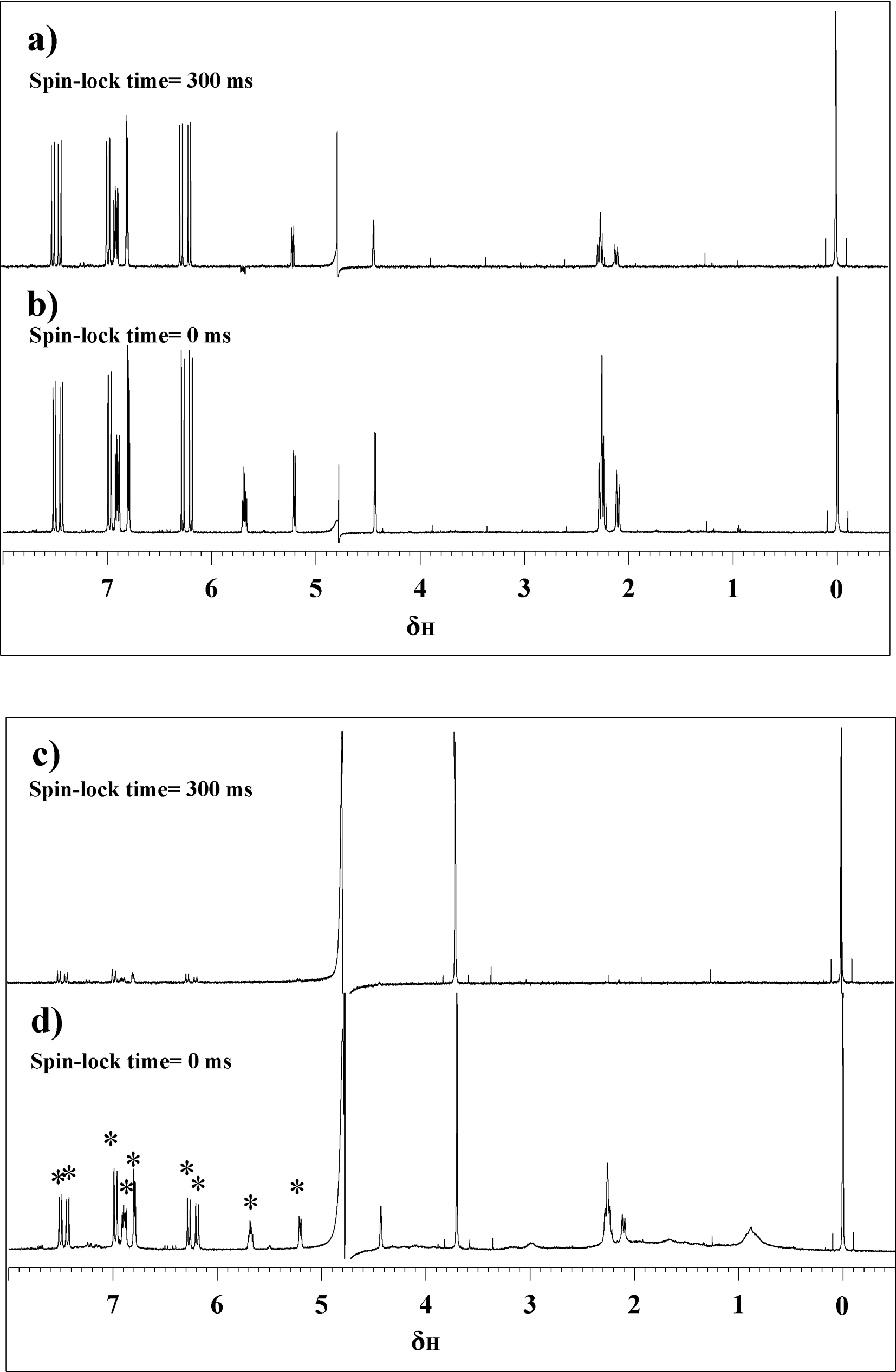

Supplement: Figure S2 — Binder screening by relaxation-edited NMR. Spectra of 4,5-dicaffeoylquinic acid (compound c) in the absence (plots a , b) and presence (plots c , d) of PAC. The CPMG spin-lock time of each experiment was labeled beside the spectrum. The concentration of the small molecule and PAC was 1.0×10^−3^ mol/L and 7.0×10^−6^ mol/L, respectively. The water peak located at δ 4.8 and 1 mM of TSP was added to the sample as a reference (δ 0). The ligand peaks that attenuated when applying CPMG spin-lock in the presence of PAC were marked with “*” in plot d. (TIF) [file pone.0035234.s002.tif]

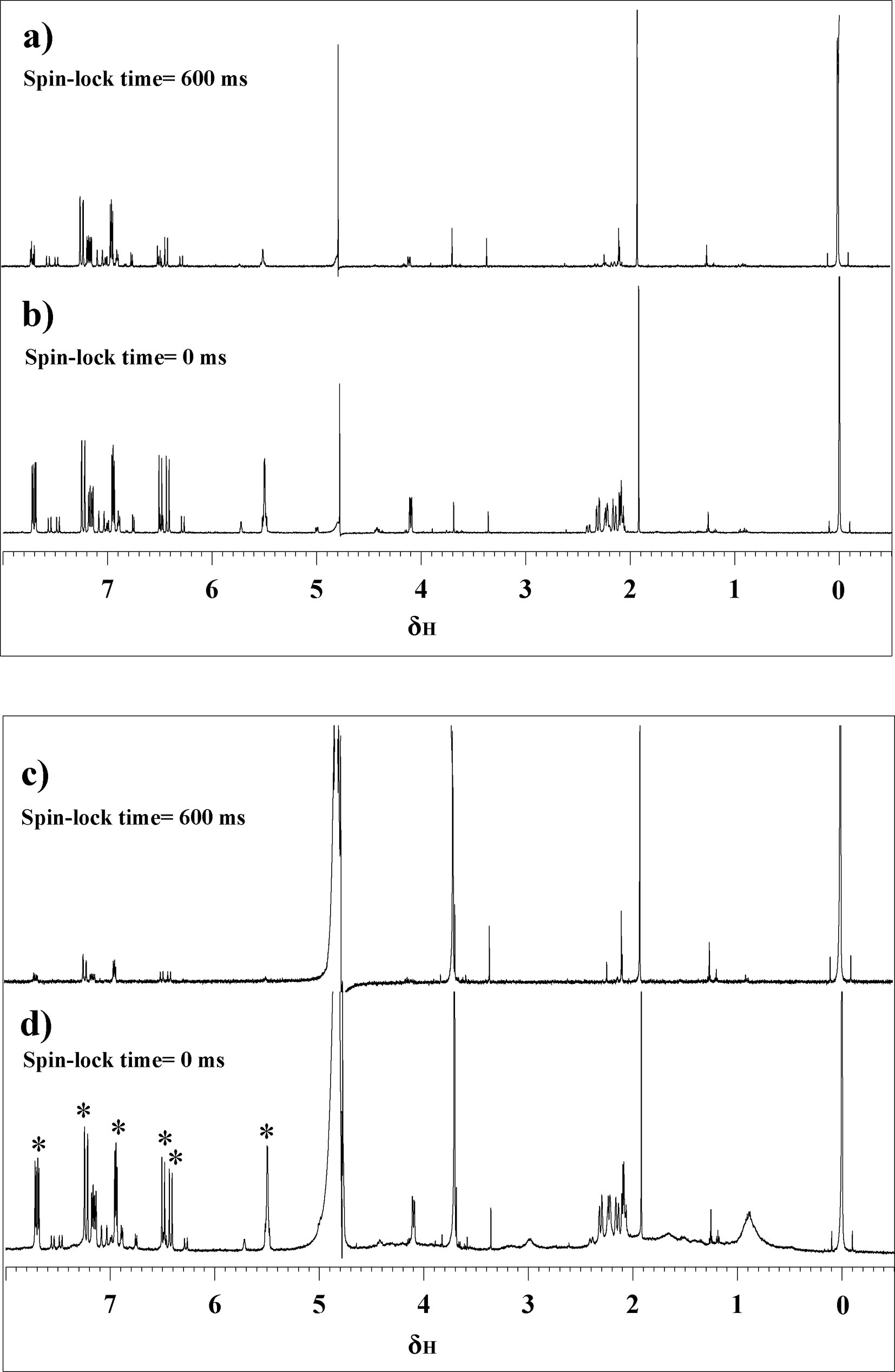

Supplement: Figure S3 — Binder screening by relaxation-edited NMR. Spectra of 3,5-dicaffeoylquinic acid (compound d) in the absence (plots a , b) and presence (plots c , d) of PAC. The CPMG spin-lock time of each experiment was labeled beside the spectrum. The concentration of the small molecule and PAC was 1.0×10^−3^ mol/L and 7.0×10^−6^ mol/L, respectively. The water peak located at δ 4.8 and 1 mM of TSP was added to the sample as a reference (δ 0). The ligand peaks that attenuated when applying CPMG spin-lock in the presence of PAC were marked with “*” in plot d. (TIF) [file pone.0035234.s003.tif]

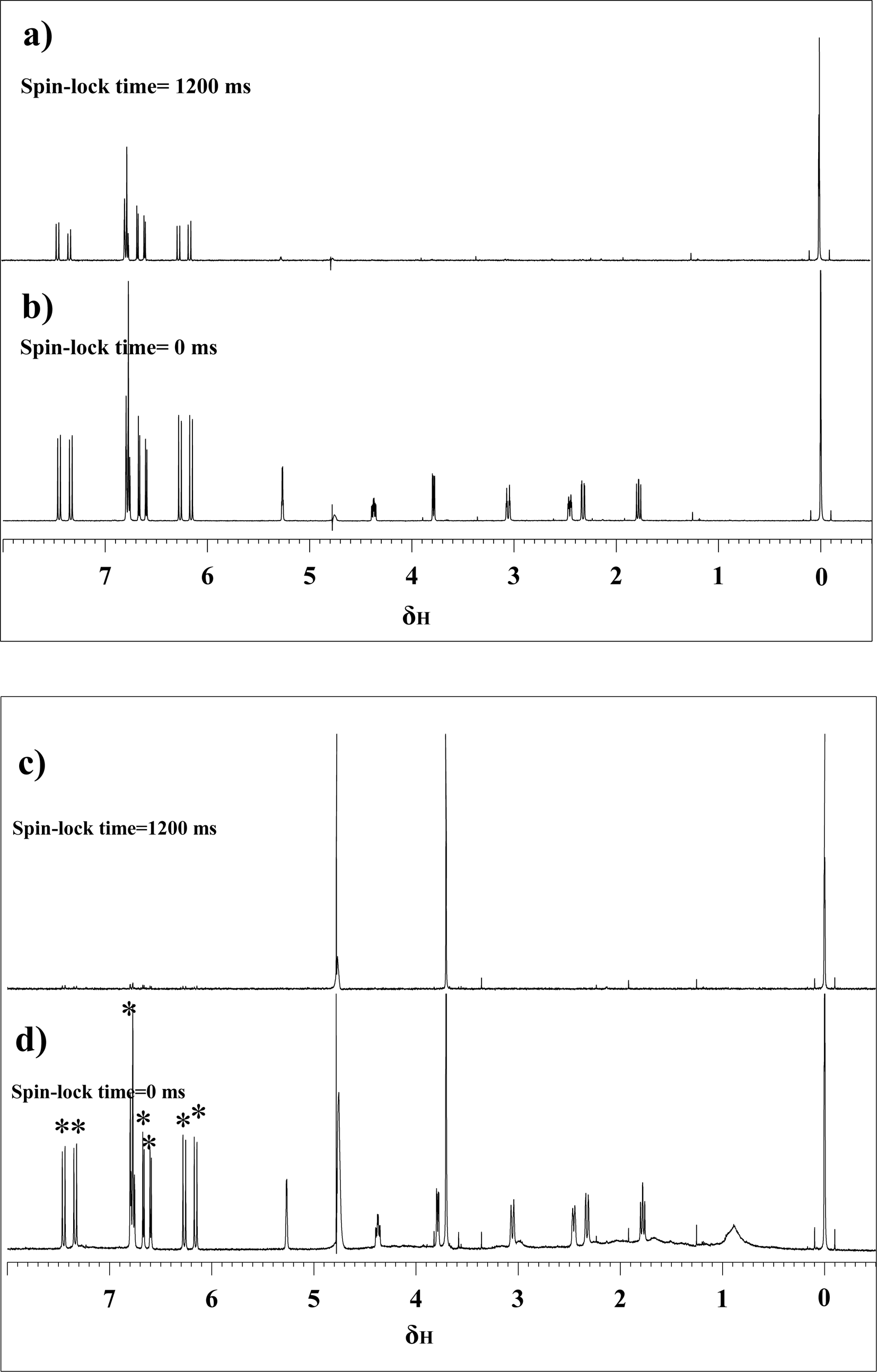

Supplement: Figure S4 — Binder screening by relaxation-edited NMR. Spectra of 1,3-dicaffeoylquinic acid (compound e) in the absence (plots a , b) and presence (plots c , d) of PAC. The CPMG spin-lock time of each experiment was labeled beside the spectrum. The concentration of the small molecule and PAC was 1.0×10^−3^ mol/L and 5.5×10^−6^ mol/L, respectively. The water peak located at δ 4.8 and 1 mM of TSP was added to the sample as a reference (δ 0). The ligand peaks that attenuated when applying CPMG spin-lock in the presence of PAC were marked with “*” in plots d. (TIF) [file pone.0035234.s004.tif]

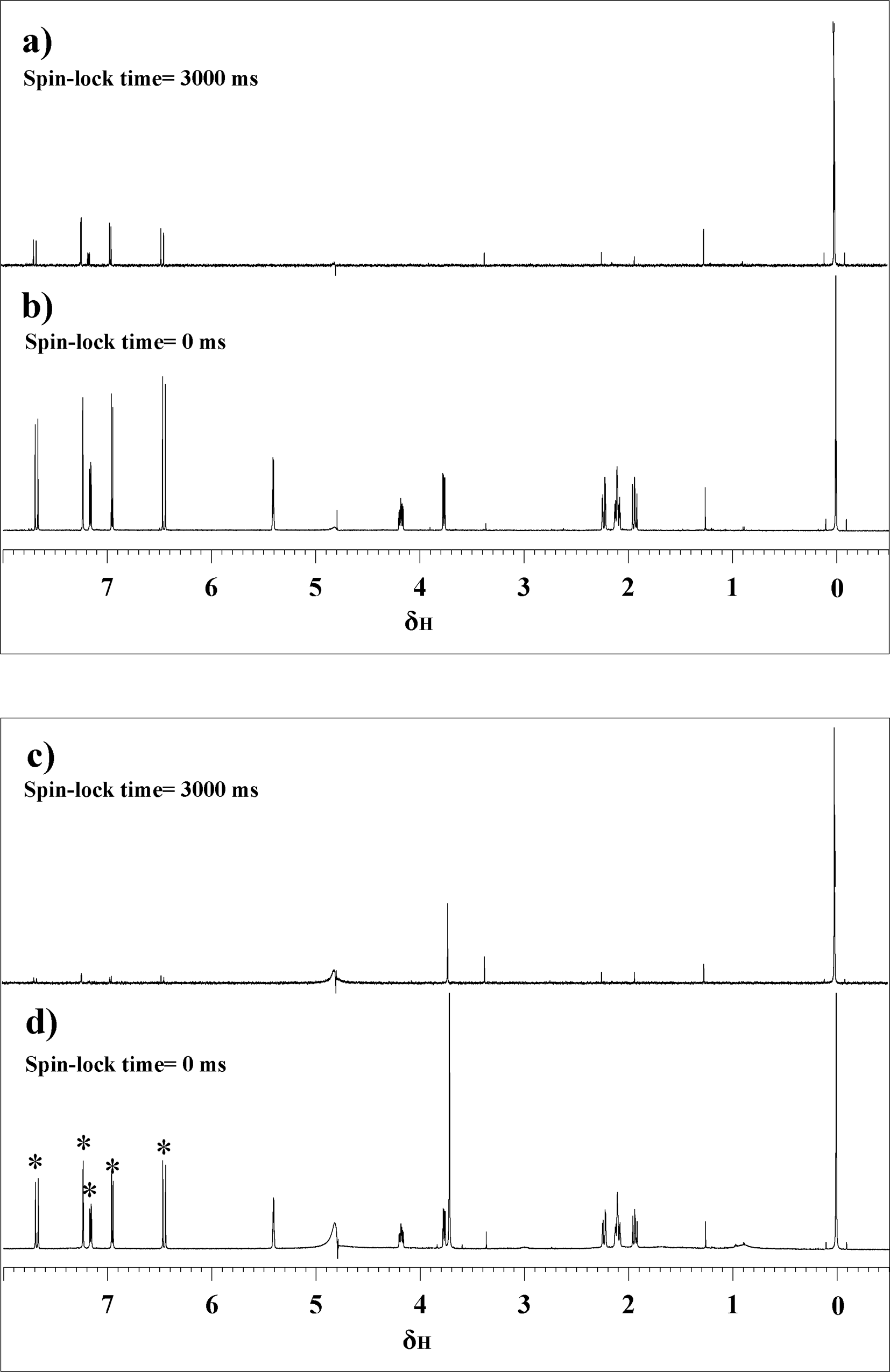

Supplement: Figure S5 — Binder screening by relaxation-edited NMR. Spectra of 5-caffeoylquinic acid (compound f) in the absence (plots a , b) and presence (plots c , d) of PAC. The CPMG spin-lock time of each experiment was labeled beside the spectrum. The concentration of the small molecule and PAC was 1.0×10^−3^ mol/L and 8.7×10^−6^ mol/L, respectively. The water peak located at δ 4.8 and 1 mM of TSP was added to the sample as a reference (δ 0). The ligand peaks that attenuated when applying CPMG spin-lock in the presence of PAC were marked with “*” in plot d. (TIF) [file pone.0035234.s005.tif]

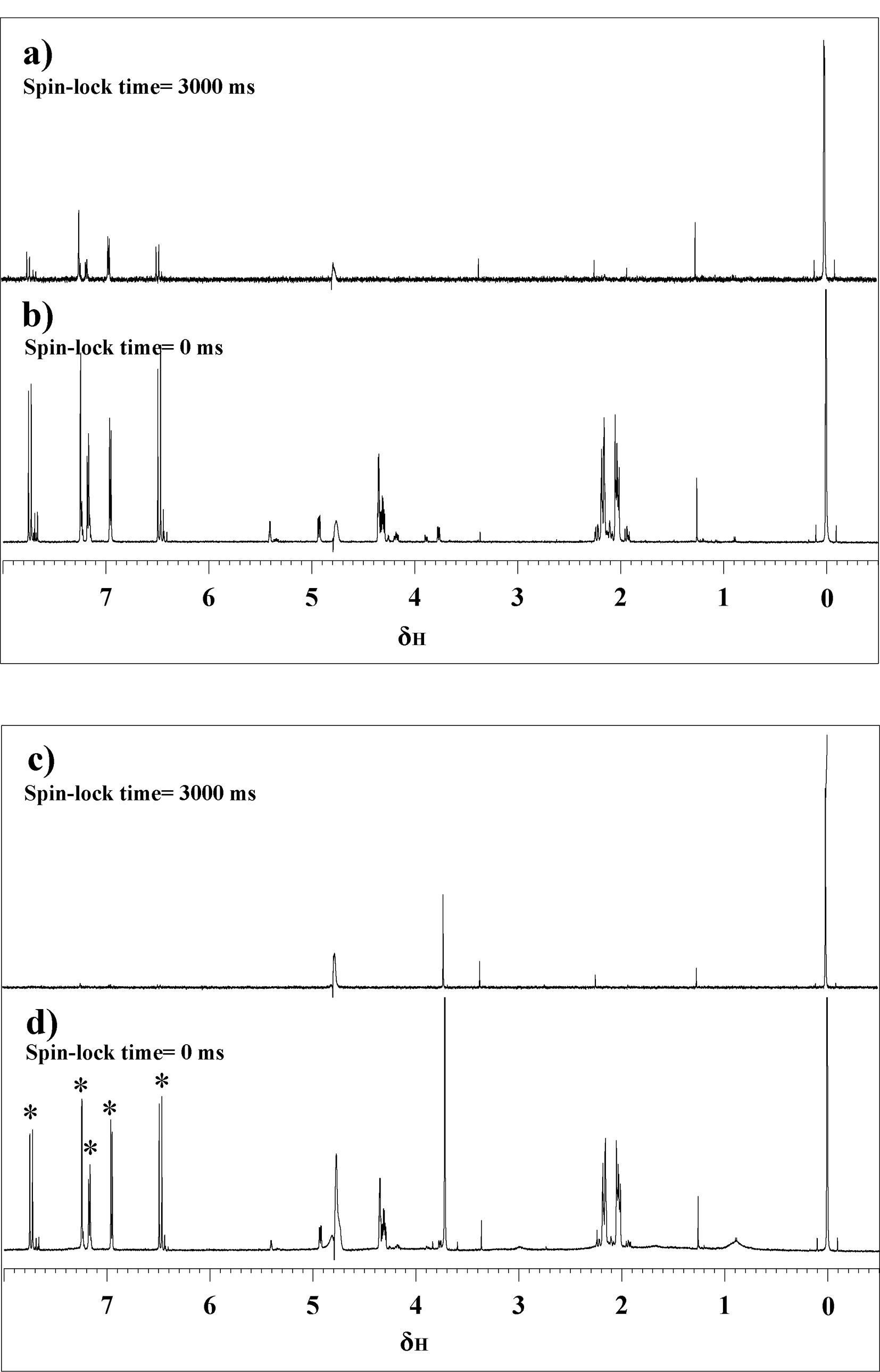

Supplement: Figure S6 — Binder screening by relaxation-edited NMR. Spectra of 4-caffeoylquinic acid (compound g) in the absence (plots a , b) and presence (plots c , d) of PAC. The CPMG spin-lock time of each experiment was labeled beside the spectrum. The concentration of the small molecule and PAC was 1.0×10^−3^ mol/L and 8.7×10^−6^ mol/L, respectively. The water peak located at δ 4.8 and 1 mM of TSP was added to the sample as a reference (δ 0). The ligand peaks that attenuated when applying CPMG spin-lock in the presence of PAC were marked with “*” in plot d. (TIF) [file pone.0035234.s006.tif]

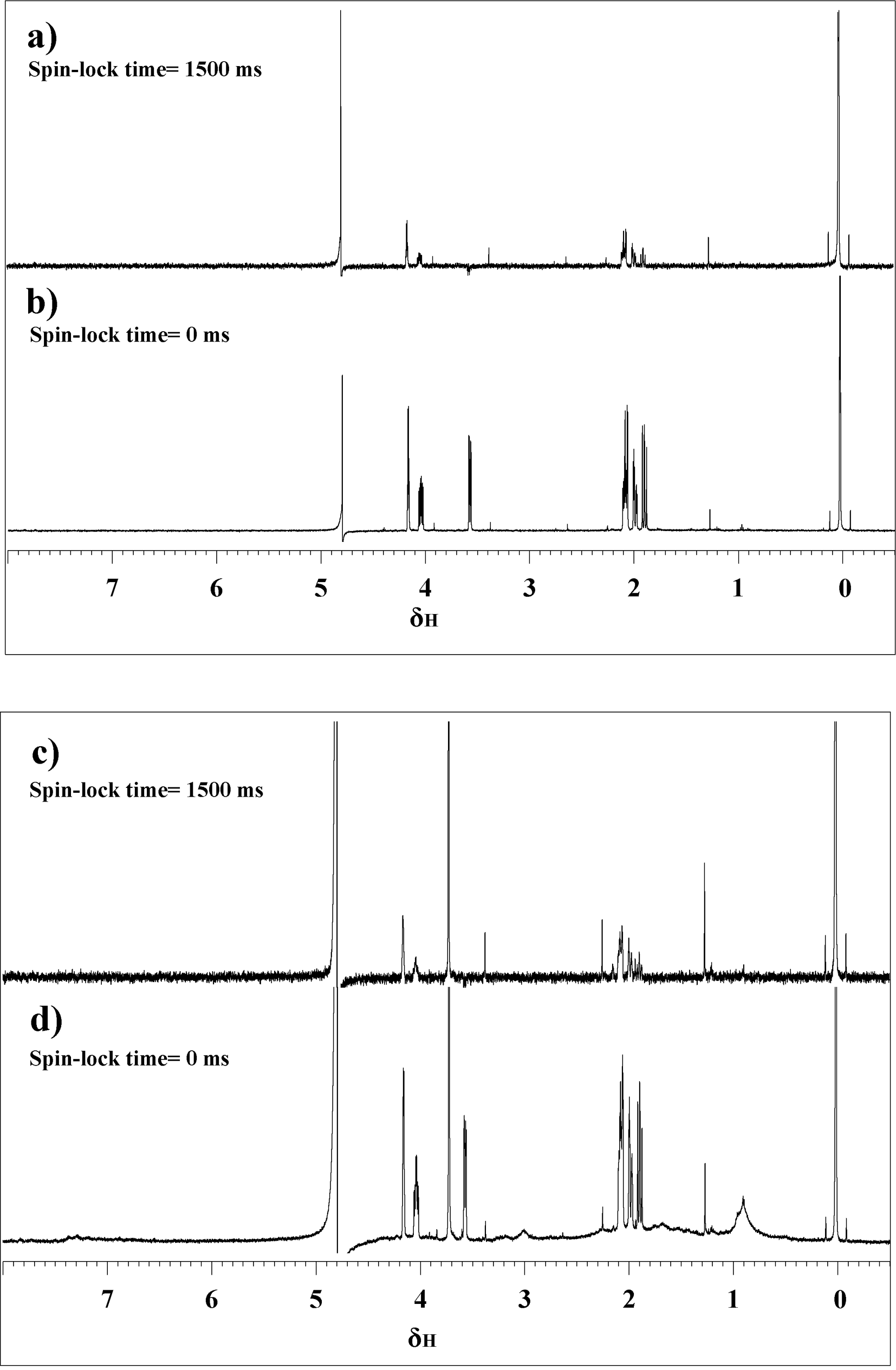

Supplement: Figure S7 — Binder screening by relaxation-edited NMR. Spectra of quinic acid (compound h) in the absence (plots a , b) and presence (plots c , d) of PAC. The CPMG spin-lock time of each experiment was labeled beside the spectrum. The concentration of the small molecule and PAC was 1.0×10^−3^ mol/L and 8.7×10^−6^ mol/L, respectively. The water peak located at δ 4.8 and 1 mM of TSP was added to the sample as a reference (δ 0). (TIF) [file pone.0035234.s007.tif]

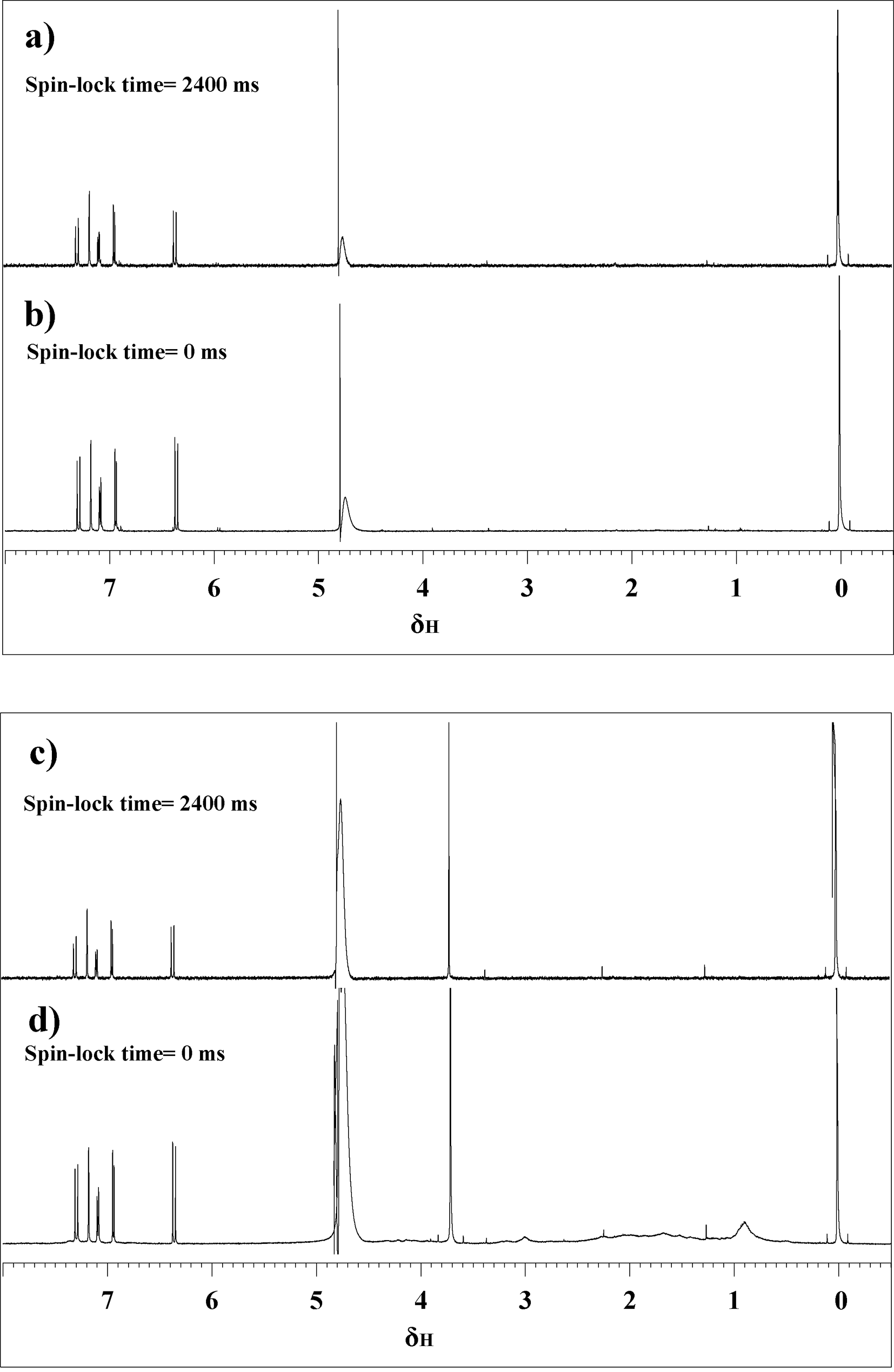

Supplement: Figure S8 — Binder screening by relaxation-edited NMR. Spectra of caffeic acid (compound i) in the absence (plots a , b) and presence (plots c , d) of PAC. The CPMG spin-lock time of each experiment was labeled beside the spectrum. The concentration of the small molecule and PAC was 1.0×10^−3^ mol/L and 8.7×10^−6^ mol/L, respectively. The water peak located at δ 4.8 and 1 mM of TSP was added to the sample as a reference (δ 0). (TIF) [file pone.0035234.s008.tif]

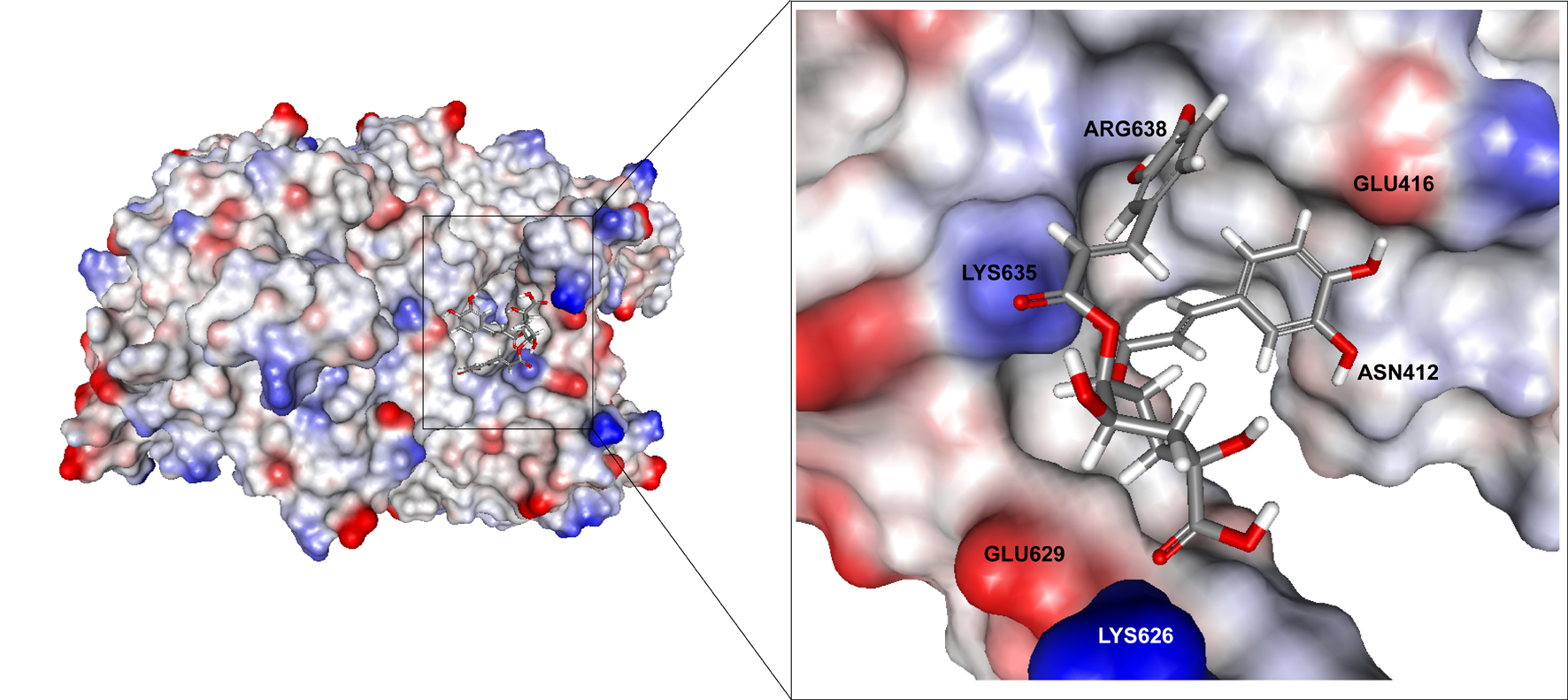

Supplement: Figure S9 — The most possible binding site of 3,4-dicaffeoylquinic acid on PAC supposed by visual docking. (TIF) [file pone.0035234.s009.tif]

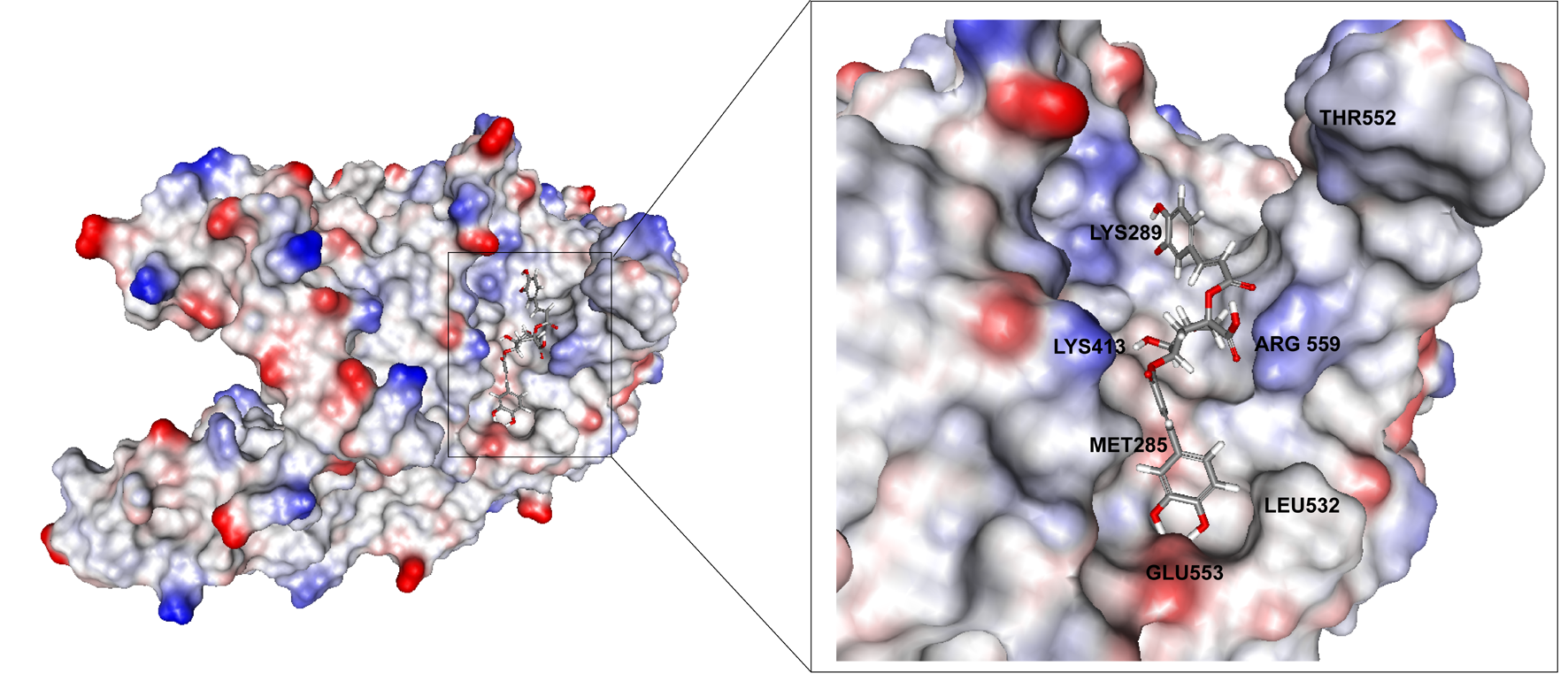

Supplement: Figure S10 — The most possible binding site of 1,5-dicaffeoylquinic acid on PAC supposed by visual docking. (TIF) [file pone.0035234.s010.tif]

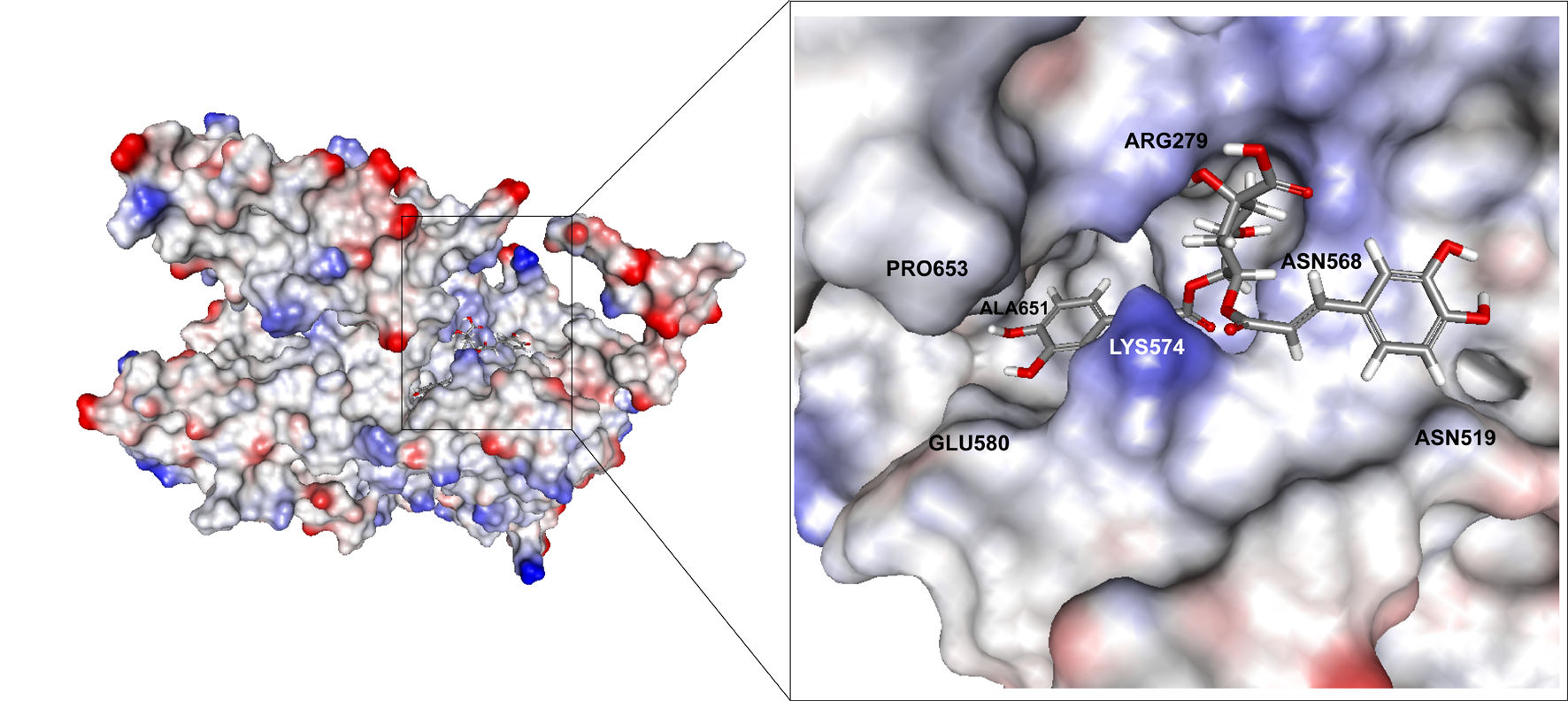

Supplement: Figure S11 — The most possible binding site of 4,5-dicaffeoylquinic acid on PAC supposed by visual docking. (TIF) [file pone.0035234.s011.tif]

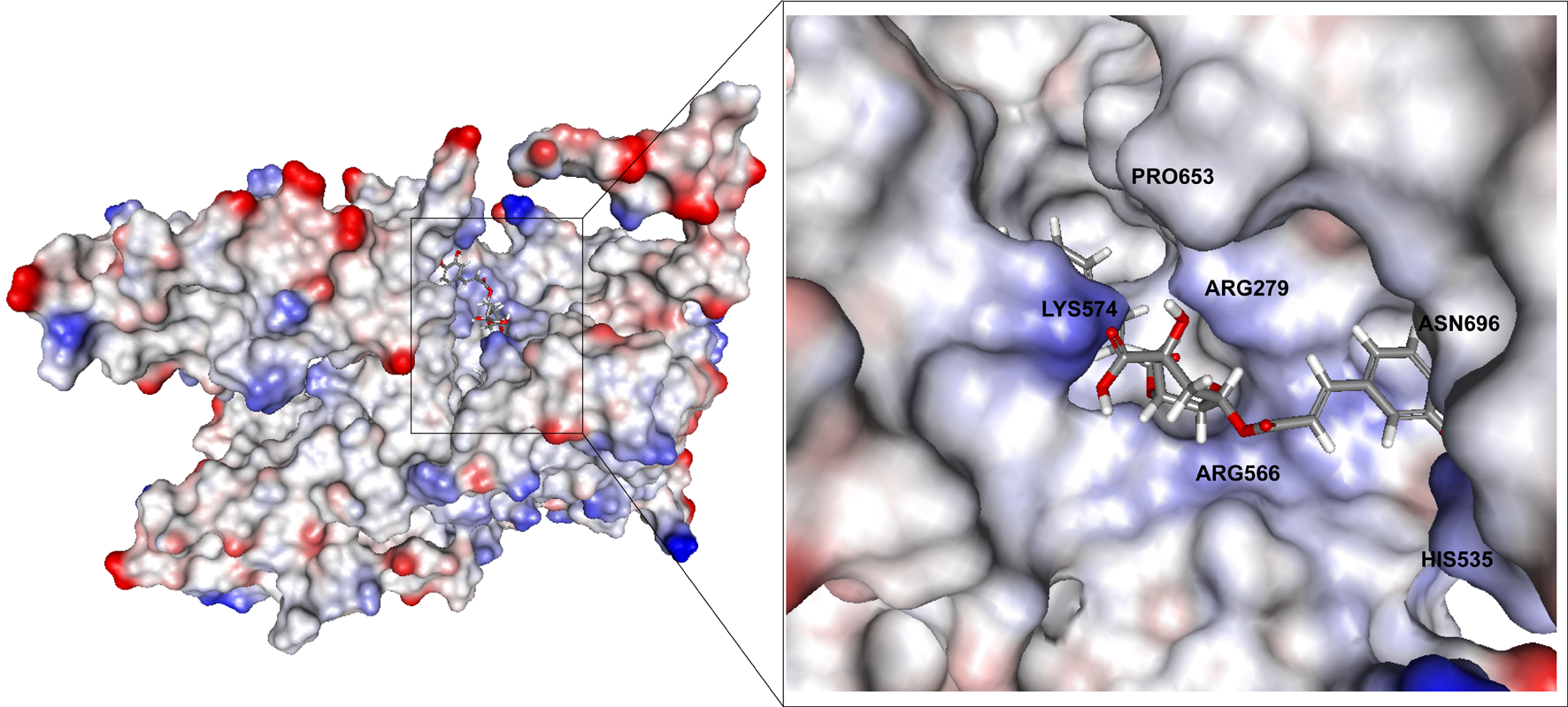

Supplement: Figure S12 — The most possible binding site of 3,5-dicaffeoylquinic acid on PAC supposed by visual docking. (TIF) [file pone.0035234.s012.tif]

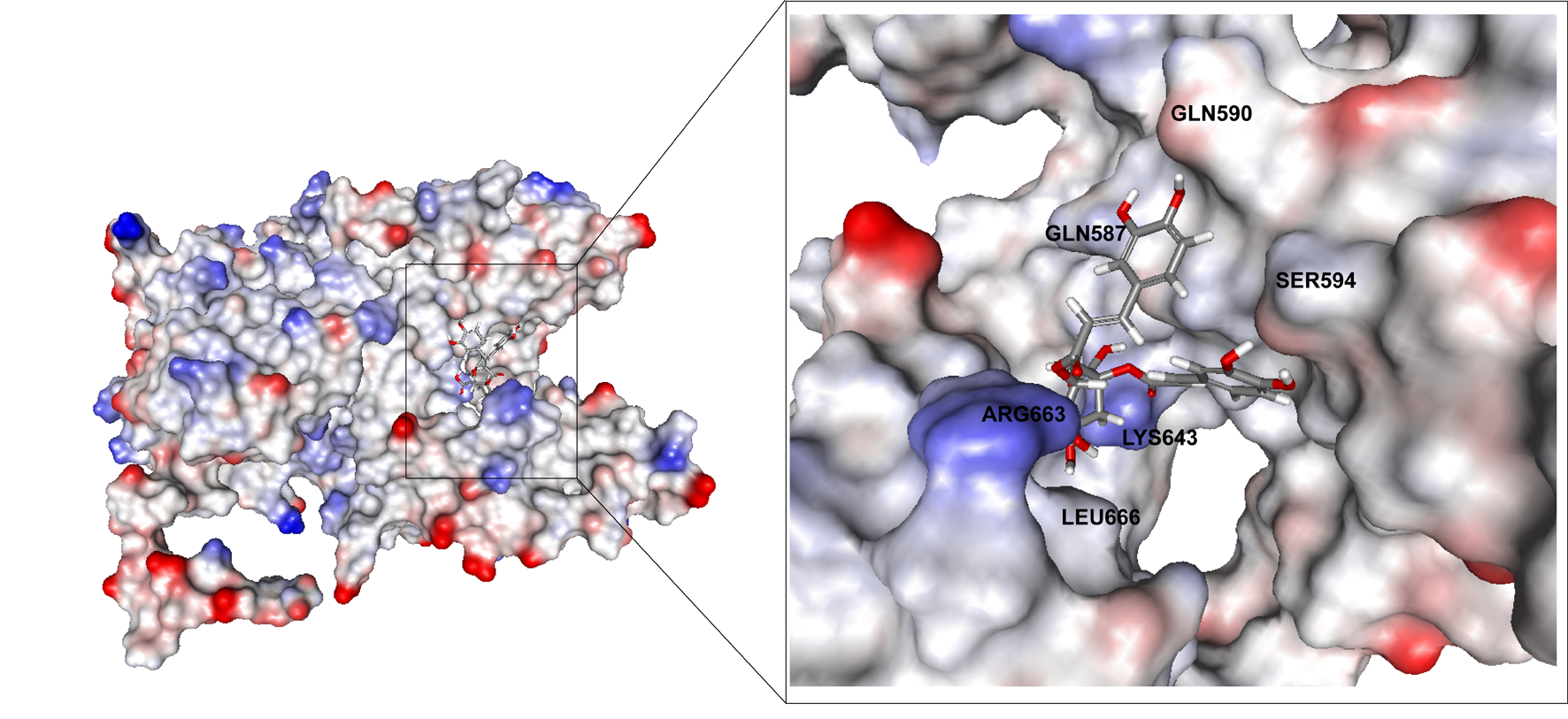

Supplement: Figure S13 — The most possible binding site of 1,3-dicaffeoylquinic acid on PAC supposed by visual docking. (TIF) [file pone.0035234.s013.tif]

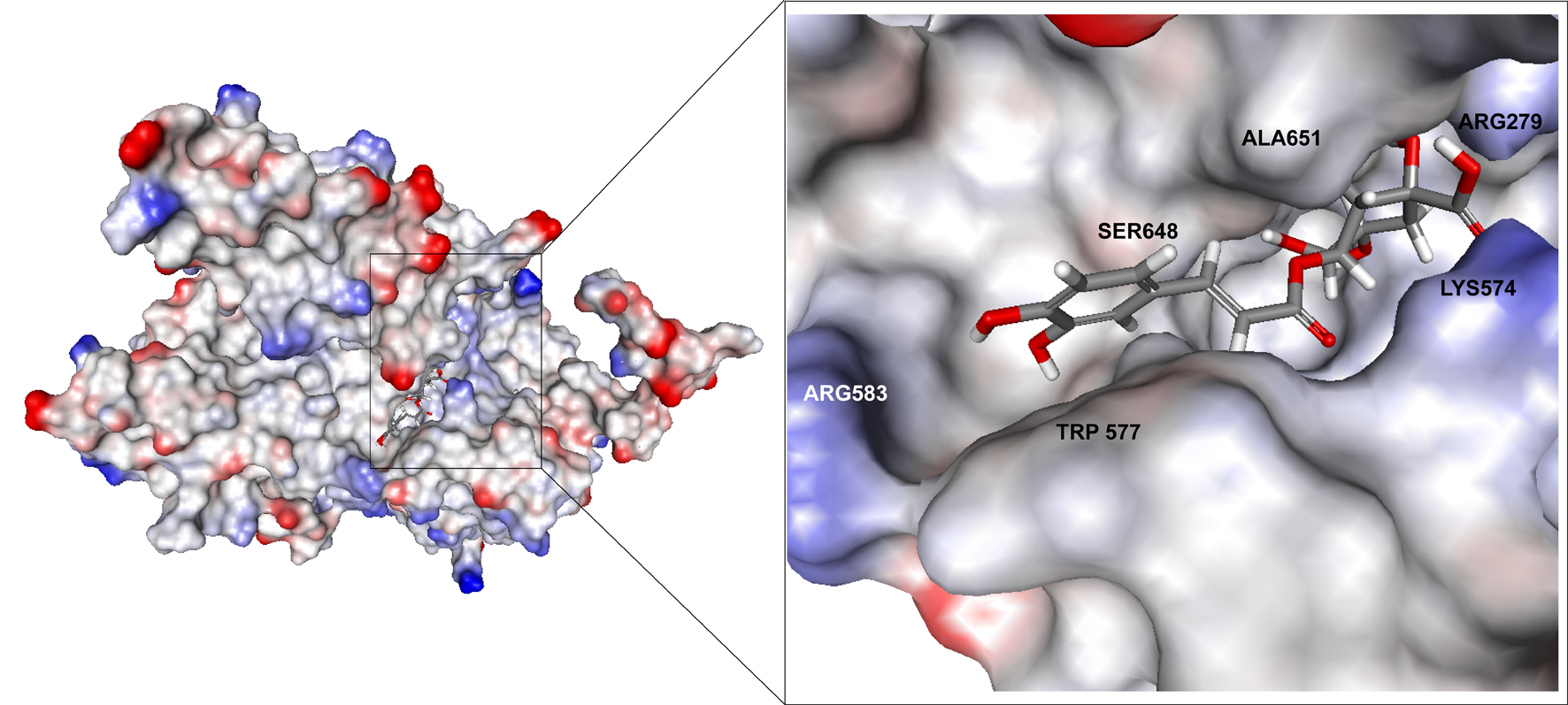

Supplement: Figure S14 — The most possible binding site of 5-caffeoylquinic acid on PAC supposed by visual docking. (TIF) [file pone.0035234.s014.tif]

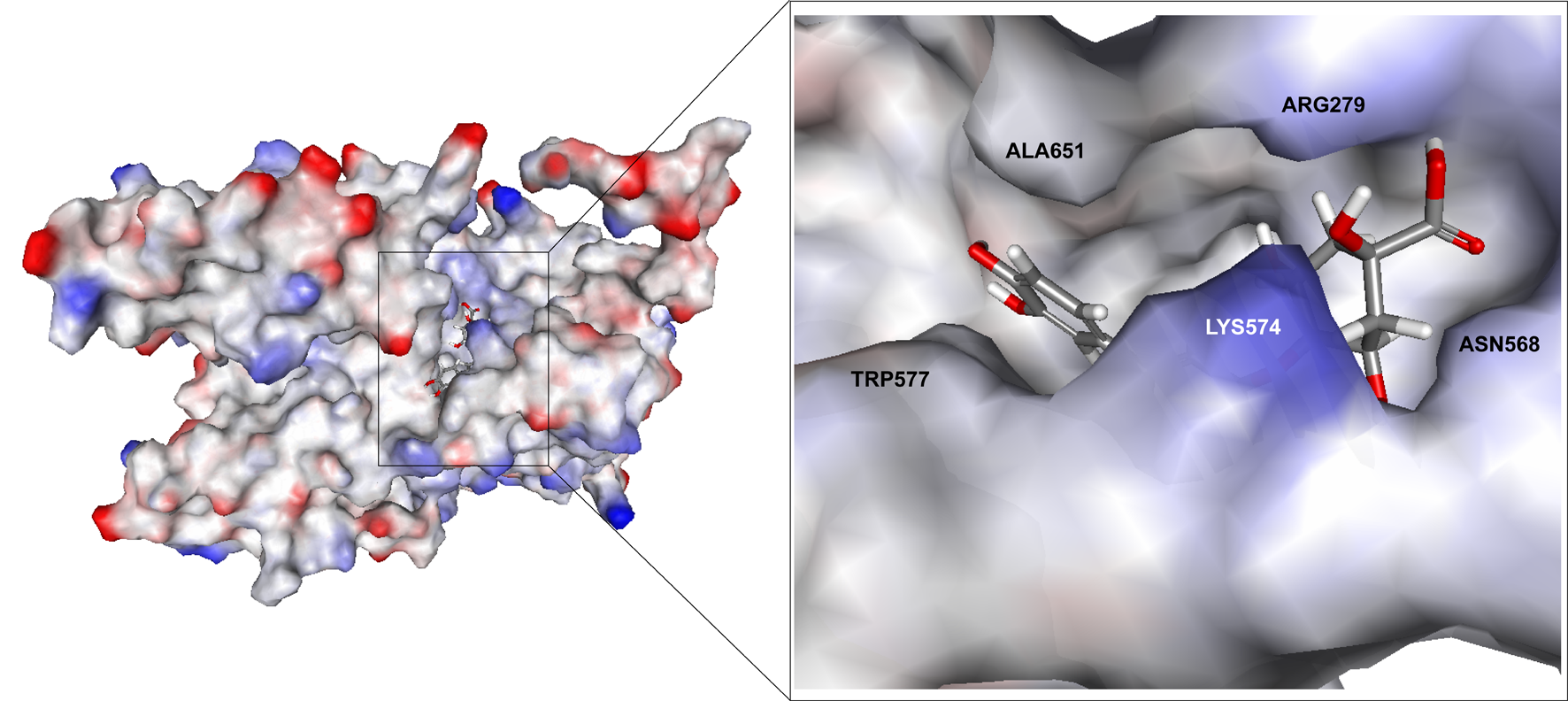

Supplement: Figure S15 — The most possible binding site of 4-caffeoylquinic acid on PAC supposed by visual docking. (TIF) [file pone.0035234.s015.tif]

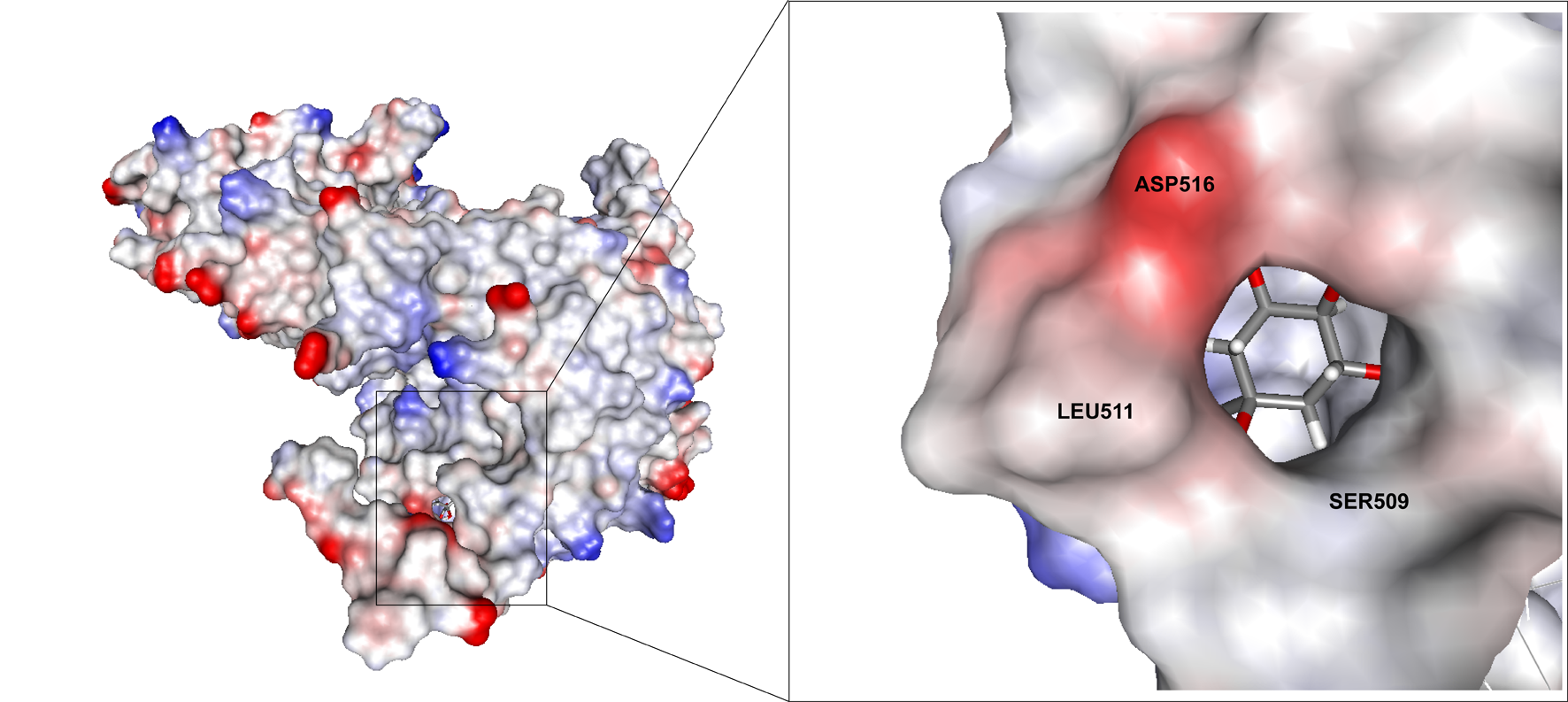

Supplement: Figure S16 — The most possible binding site of quinic acid on PAC supposed by visual docking. (TIF) [file pone.0035234.s016.tif]

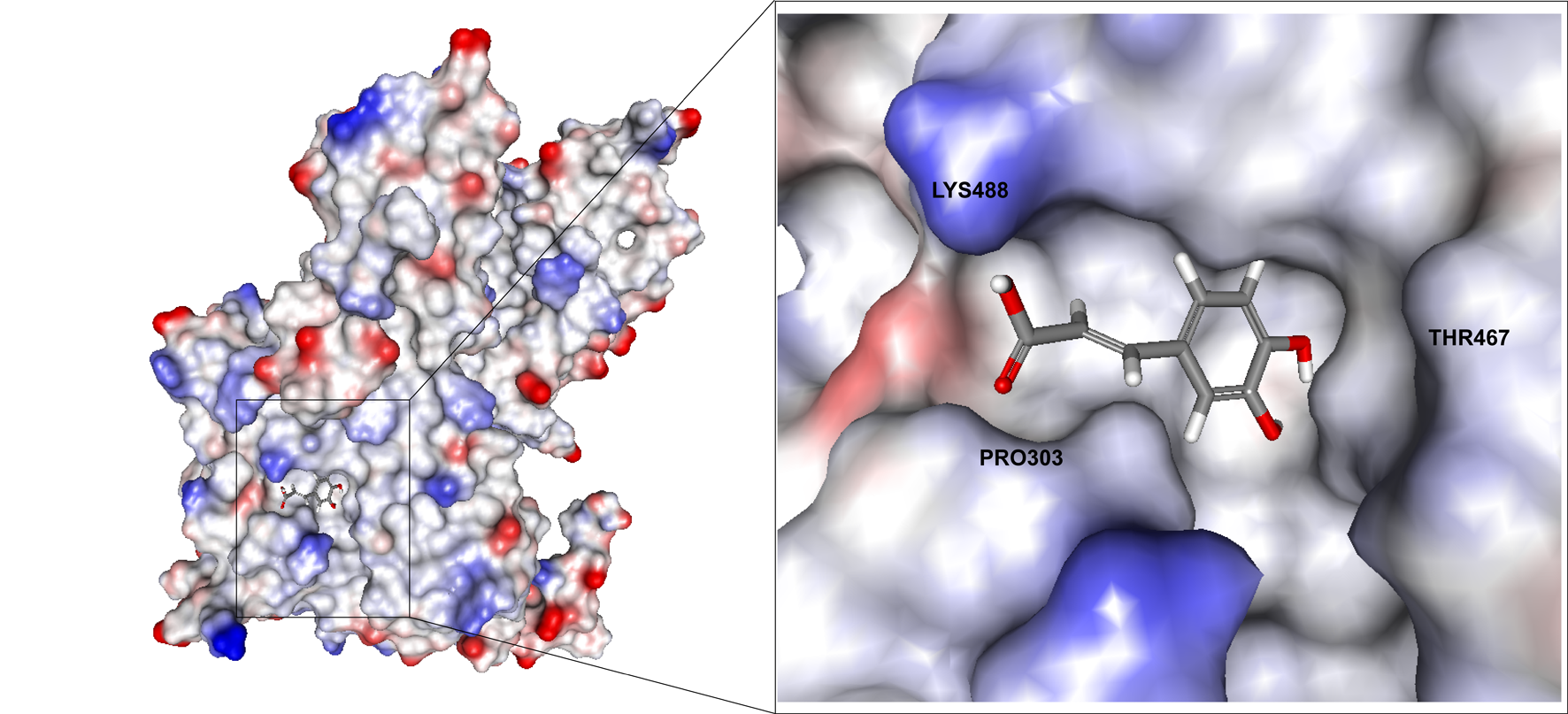

Supplement: Figure S17 — The most possible binding site of caffeic acid on PAC supposed by visual docking. (TIF) [file pone.0035234.s017.tif]

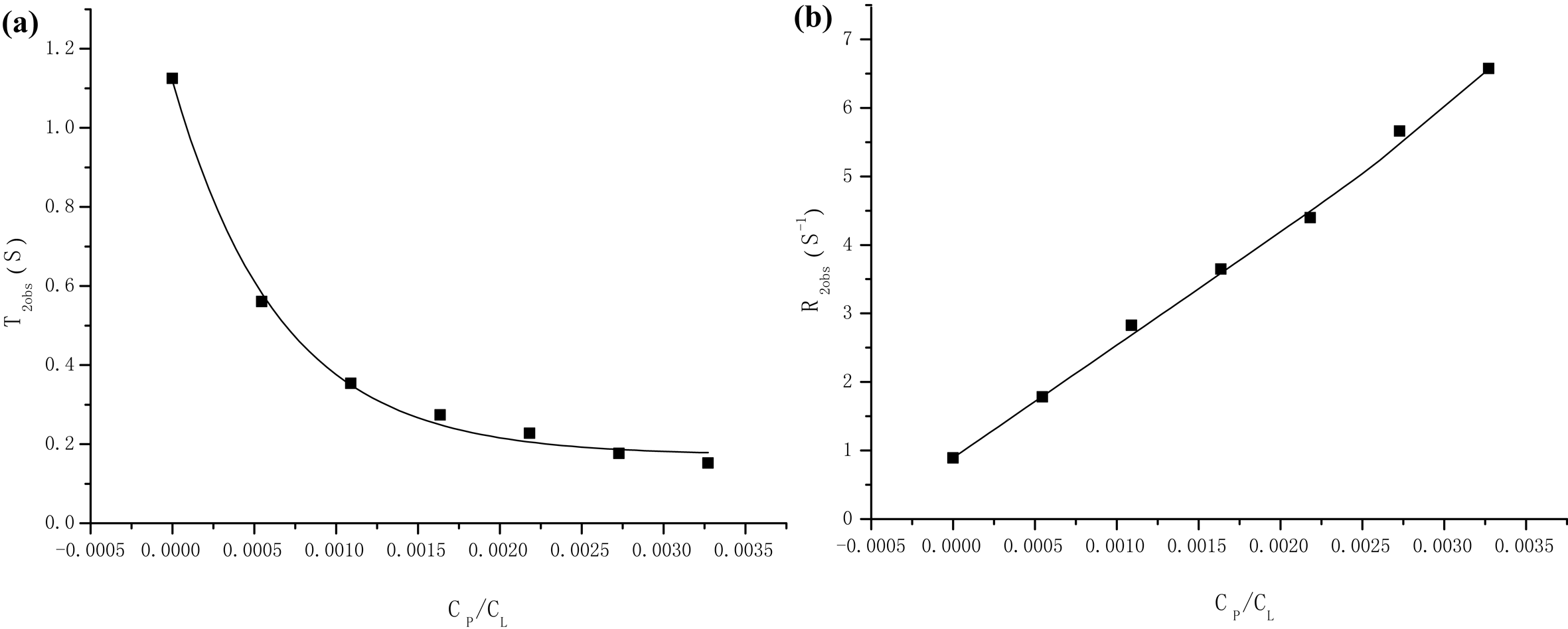

Supplement: Figure S18 — Binding affinity between 1,5-dicaffeoylquinic acid and PAC evaluated by transverse relaxation simulation. a) The plot of T 2obs versus C P/C L; b) The plot of R 2obs versus C P/C L. (TIF) [file pone.0035234.s018.tif]

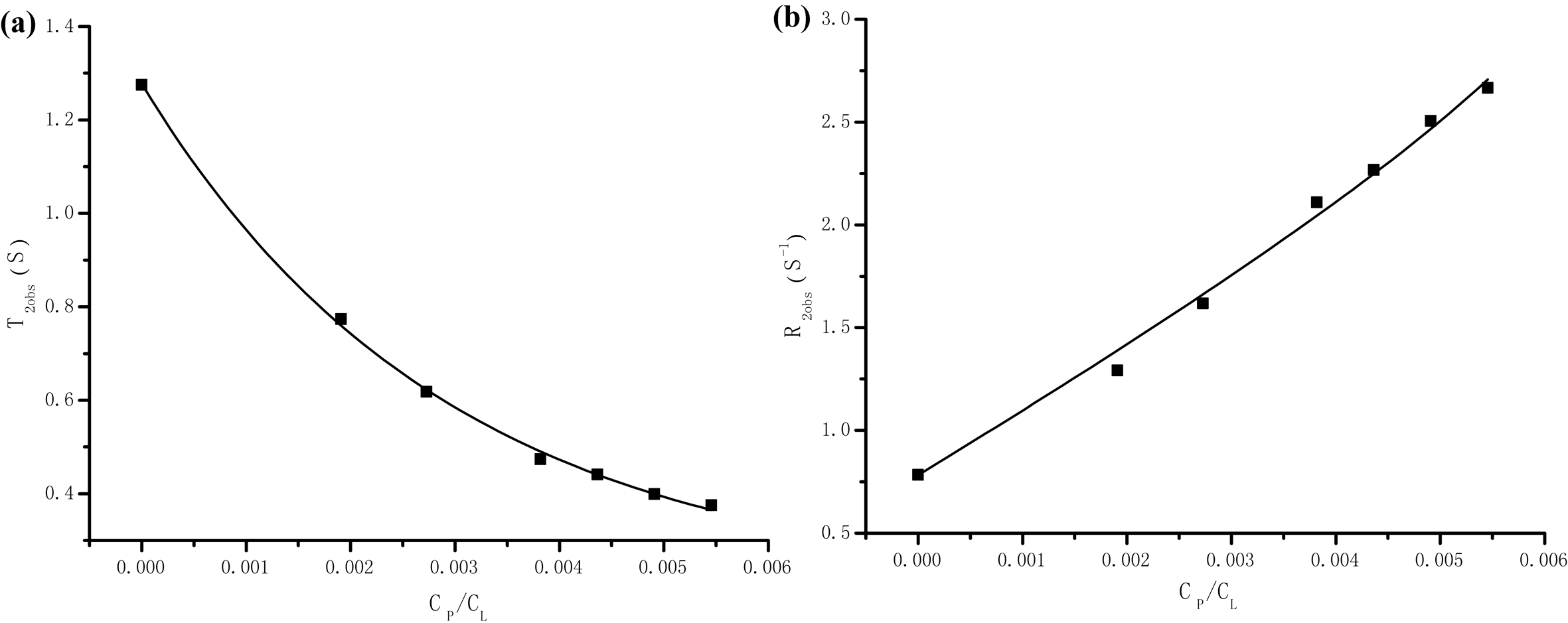

Supplement: Figure S19 — Binding affinity between 1,3-dicaffeoylquinic acid and PAC evaluated by transverse relaxation simulation. a) The plot of T 2obs versus C P/C L; b) The plot of R 2obs versus C P/C L. (TIF) [file pone.0035234.s019.tif]

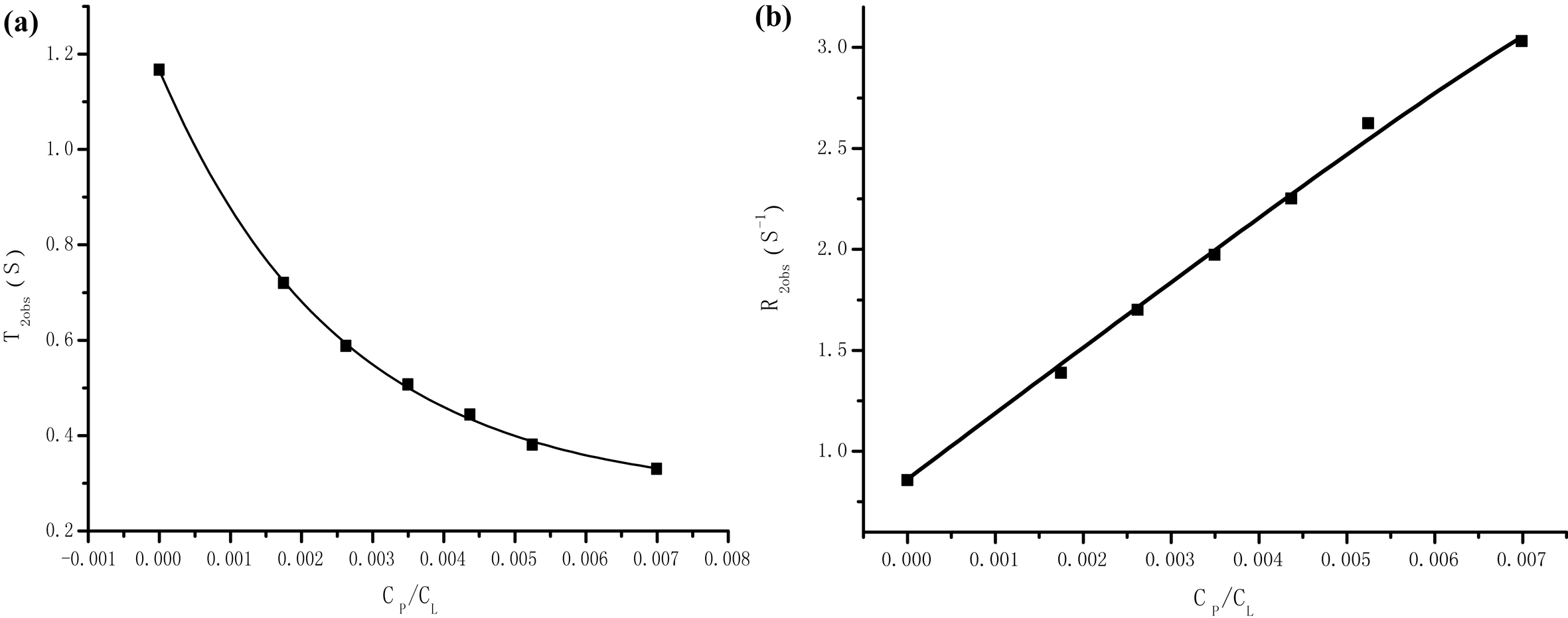

Supplement: Figure S20 — Binding affinity between 3,5-dicaffeoylquinic acid and PAC evaluated by transverse relaxation simulation. a) The plot of T 2obs versus C P/C L; b) The plot of R 2obs versus C P/C L. (TIF) [file pone.0035234.s020.tif]

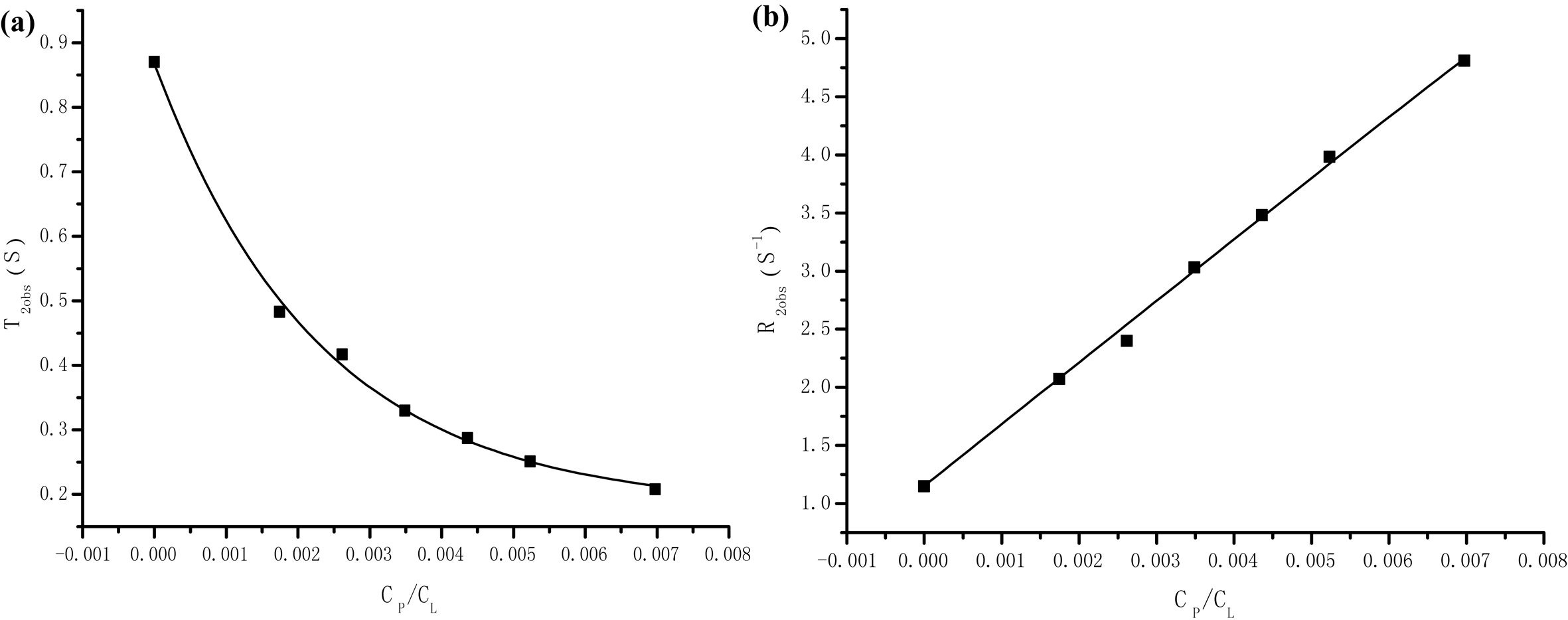

Supplement: Figure S21 — Binding affinity between 3,4-dicaffeoylquinic acid and PAC evaluated by transverse relaxation simulation. a) The plot of T 2obs versus C P/C L; b) The plot of R 2obs versus C P/C L. (TIF) [file pone.0035234.s021.tif]

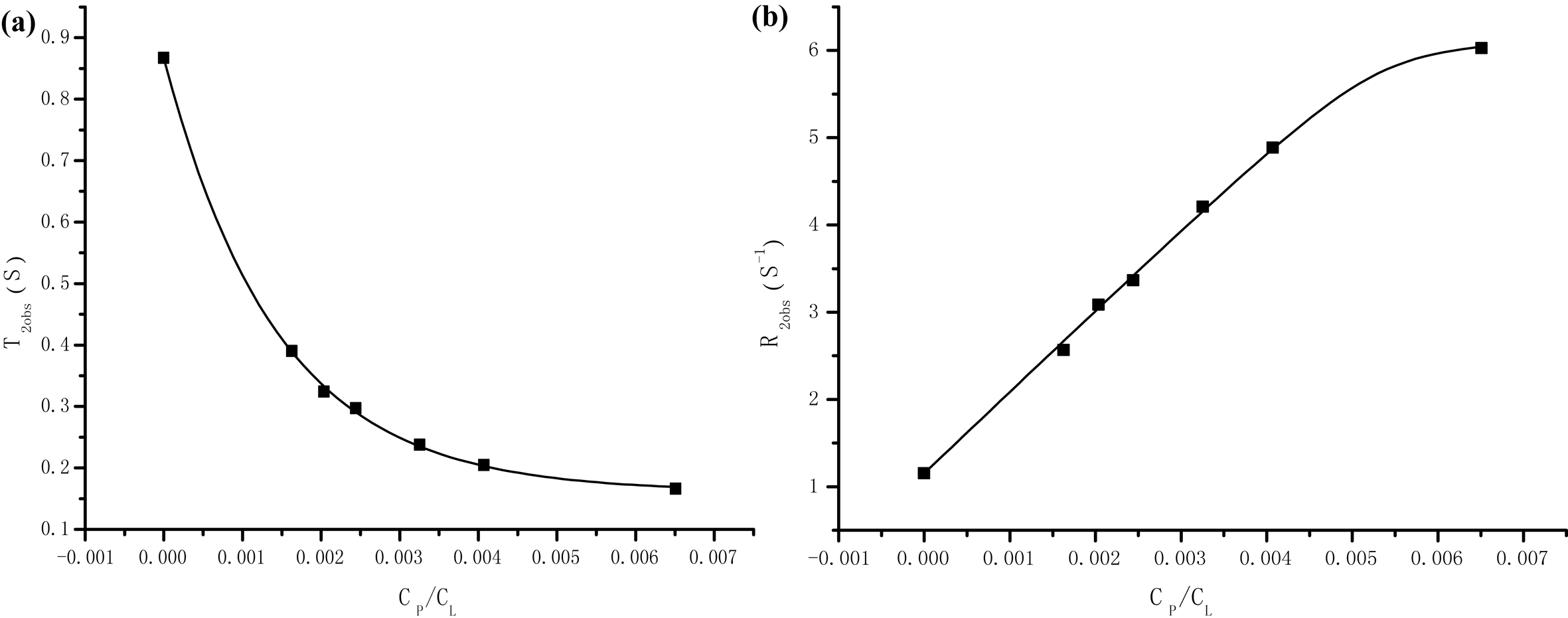

Supplement: Figure S22 — Binding affinity between 4,5-dicaffeoylquinic acid and PAC evaluated by transverse relaxation simulation. a) The plot of T 2obs versus C P/C L; b) The plot of R 2obs versus C P/C L. (TIF) [file pone.0035234.s022.tif]

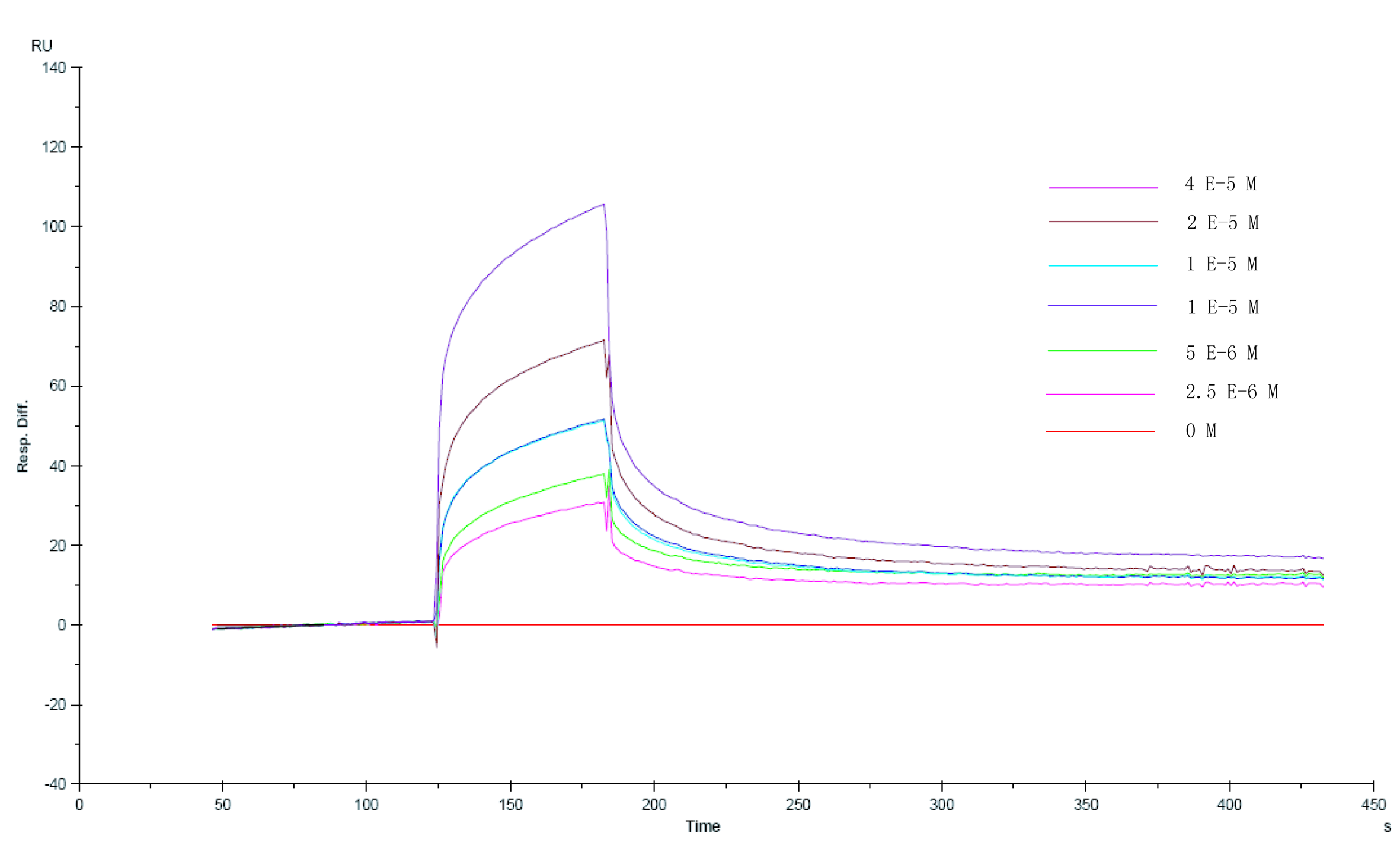

Supplement: Figure S23 — Association and dissociation kinetics plot of 3,4-dicaffeoylquinic acid to PAC as determined by SPR. (TIF) [file pone.0035234.s023.tif]

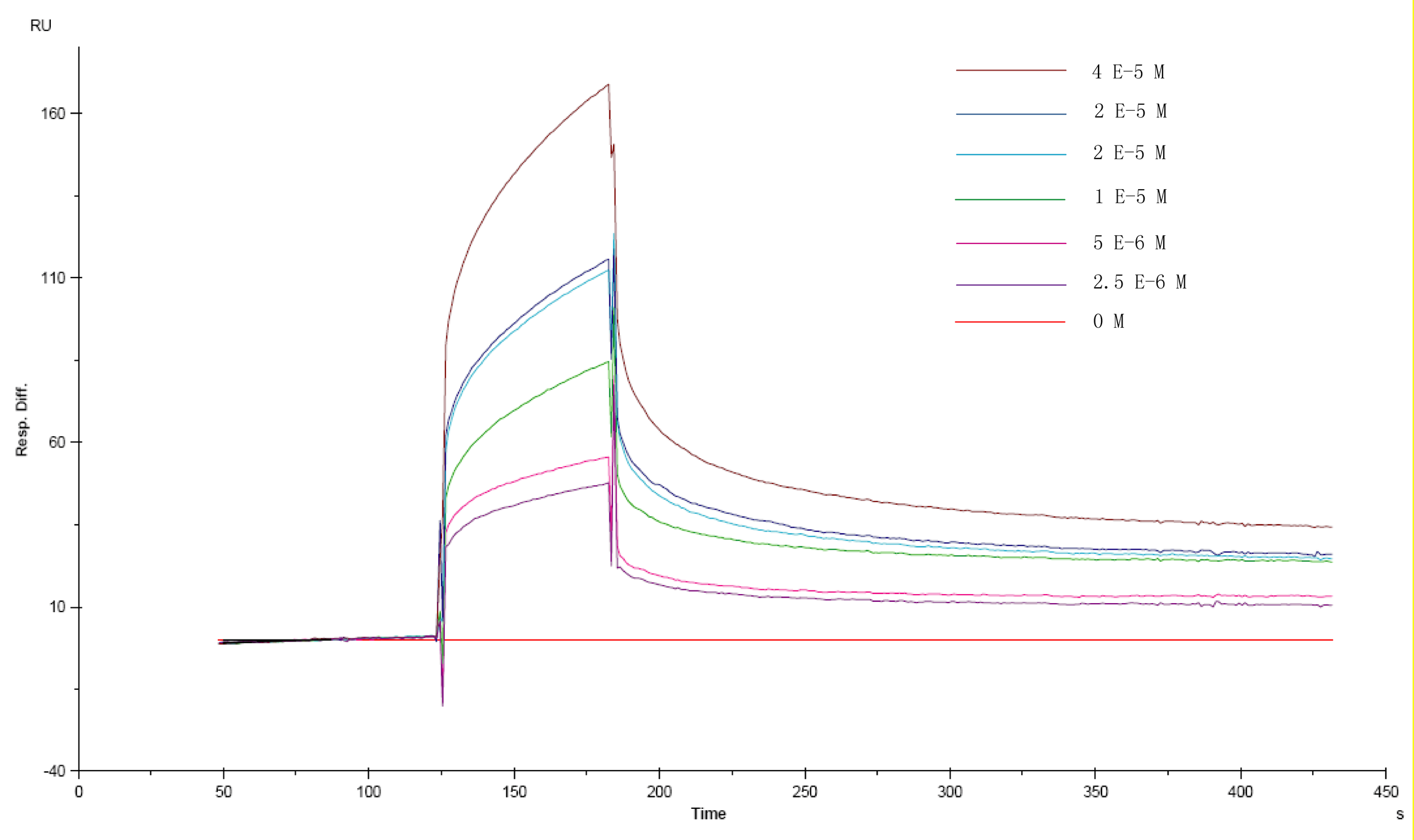

Supplement: Figure S24 — Association and dissociation kinetics plot of 1,5-dicaffeoylquinic acid to PAC as determined by SPR. (TIF) [file pone.0035234.s024.tif]

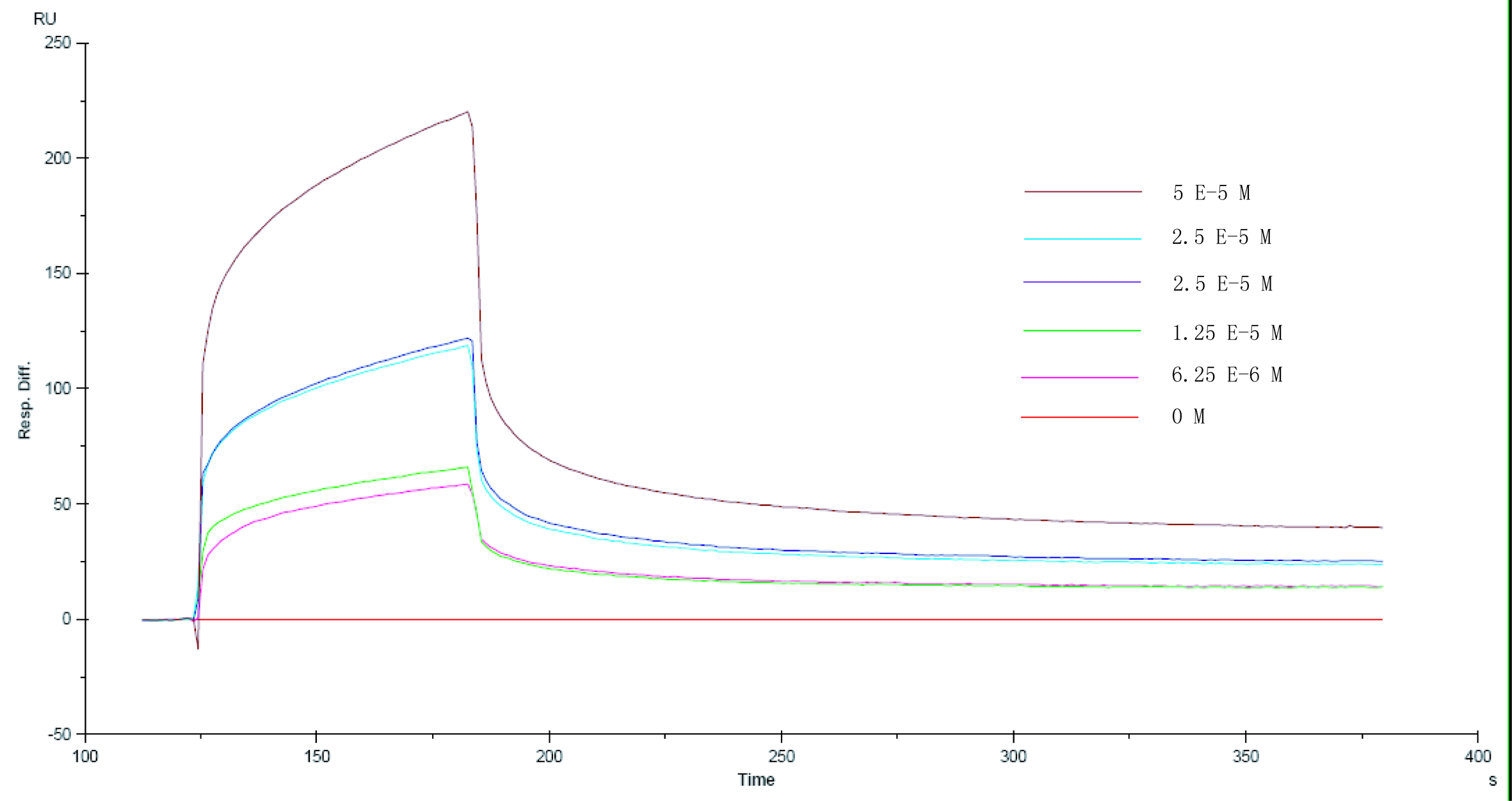

Supplement: Figure S25 — Association and dissociation kinetics plot of 4,5-dicaffeoylquinic acid to PAC as determined by SPR. (TIF) [file pone.0035234.s025.tif]

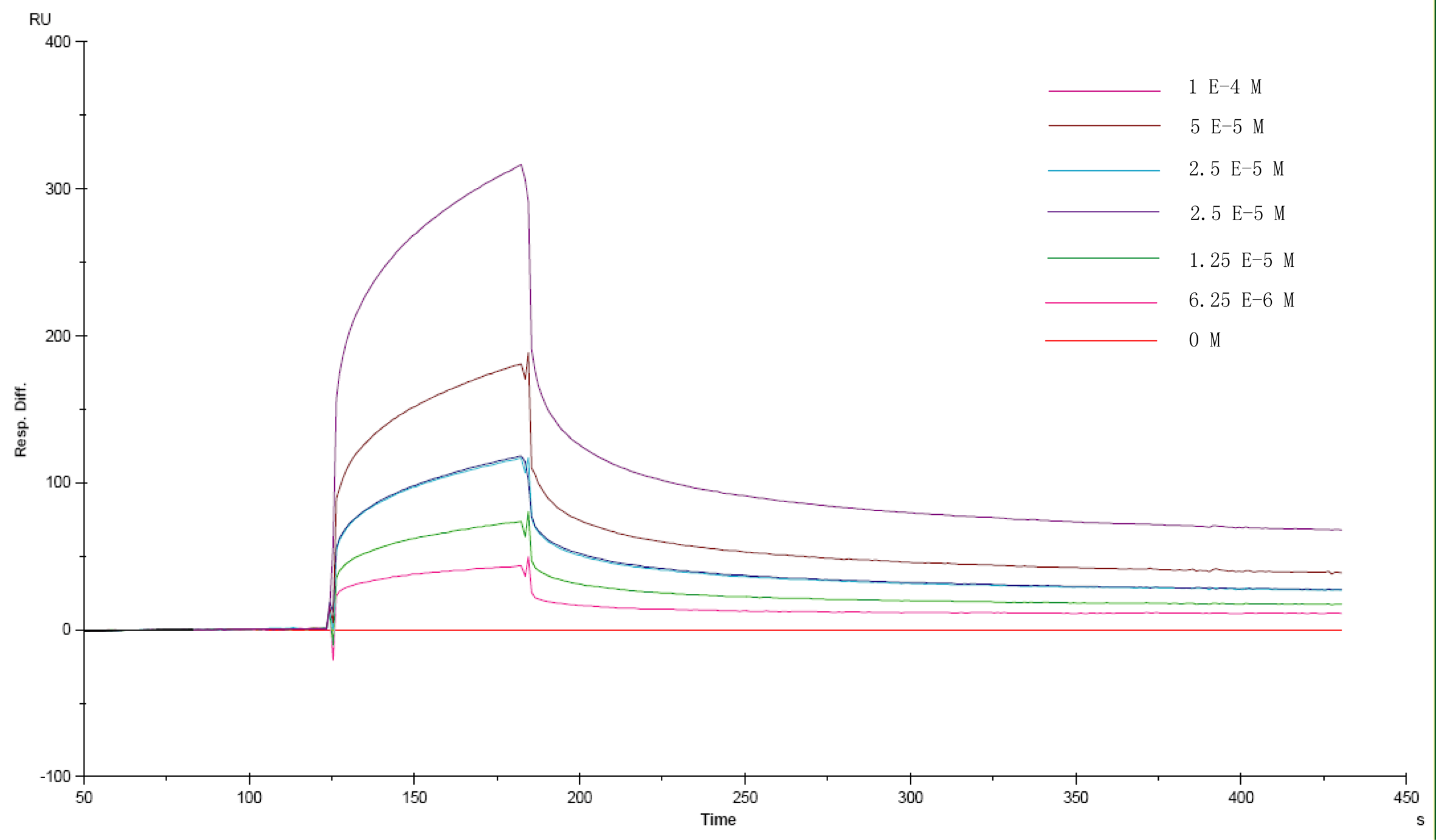

Supplement: Figure S26 — Association and dissociation kinetics plot of 3,5-dicaffeoylquinic acid to PAC as determined by SPR. (TIF) [file pone.0035234.s026.tif]

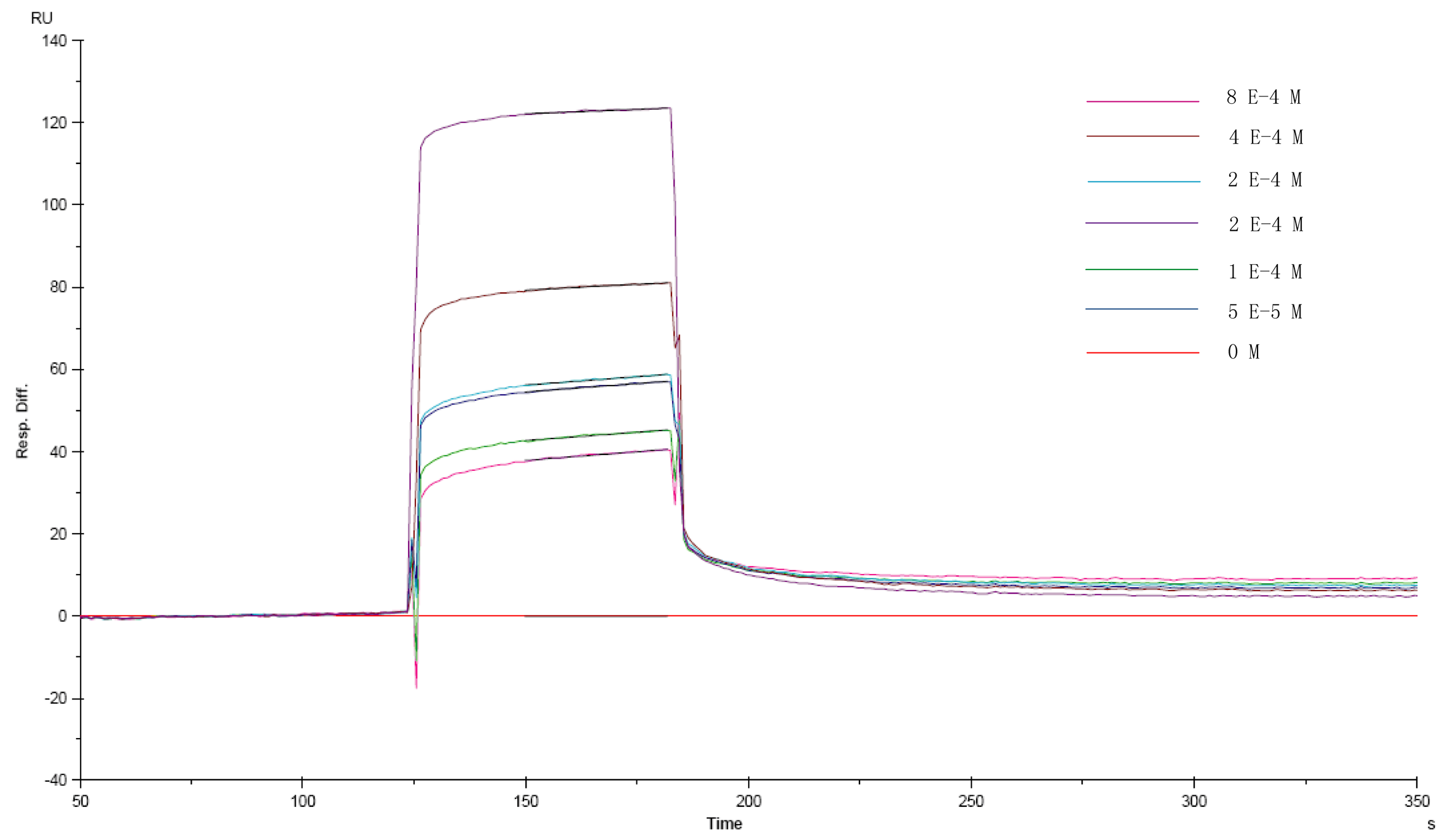

Supplement: Figure S27 — Association and dissociation kinetics plot of 1,3-dicaffeoylquinic acid to PAC as determined by SPR. (TIF) [file pone.0035234.s027.tif]

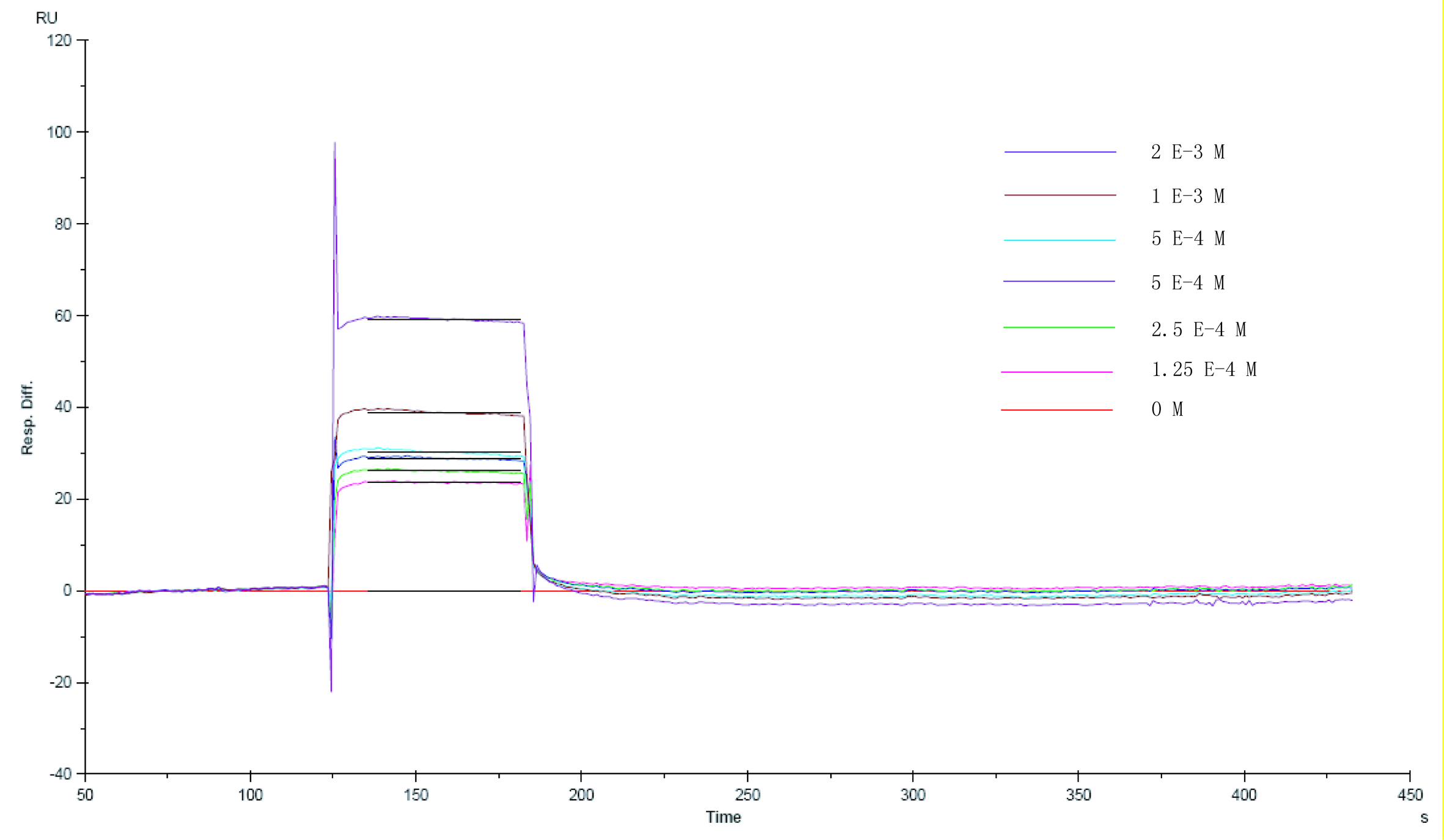

Supplement: Figure S28 — Association and dissociation kinetics plot of 5-caffeoylquinic acid to PAC as determined by SPR. (TIF) [file pone.0035234.s028.tif]

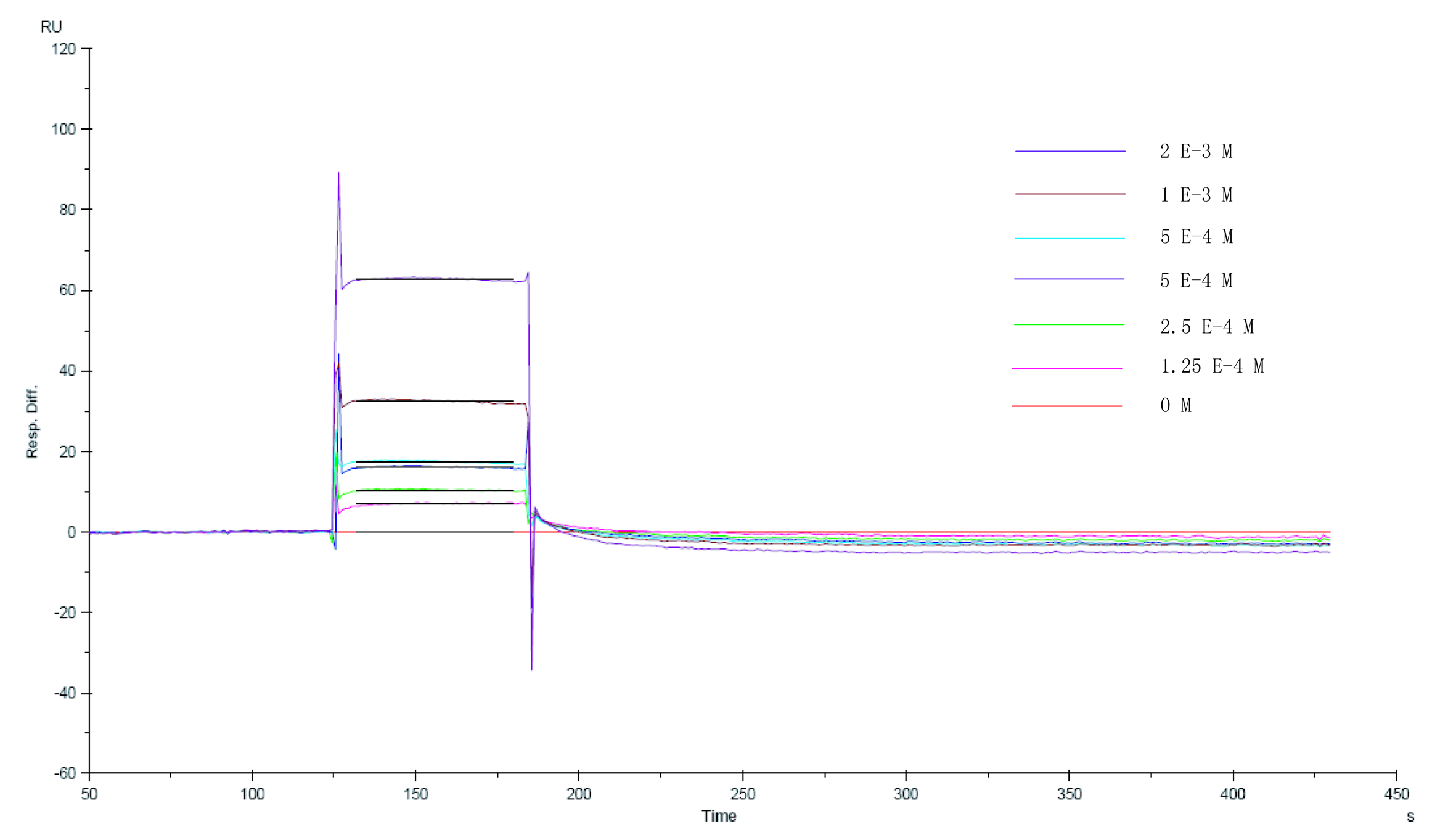

Supplement: Figure S29 — Association and dissociation kinetics plot of 4-caffeoylquinic acid to PAC as determined by SPR. (TIF) [file pone.0035234.s029.tif]

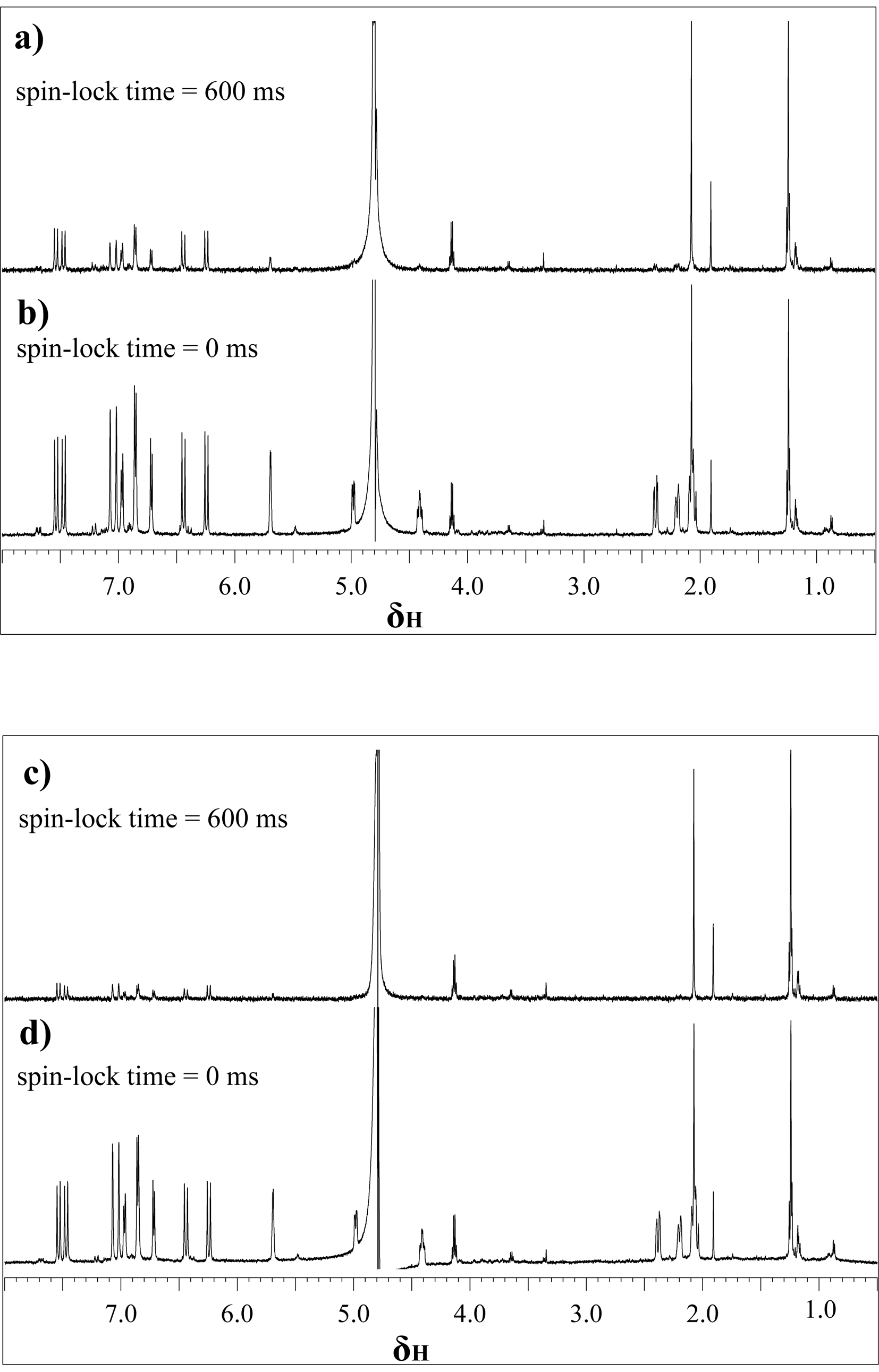

Supplement: Figure S30 — Binder screening by relaxation-edited NMR. Spectra of 3,4-dicaffeoylquinic acid (compound a) in the absence (plots a , b) and presence (plots c , d) of HSA. The CPMG spin-lock time of each experiment was labeled beside the spectra. The concentrations of small molecule and HSA were 1.0×10^−3^ mol/L and 7.0×10^−6^ mol/L, respectively. The water peak located at δ 4.8. The peaks of small molecule were not eliminated at a long spin-lock time, indicated that compound a did not specifically interact with HSA. (TIF) [file pone.0035234.s030.tif]
