# Supplementary material for: Screen Anti-influenza Lead Compounds That Target the PAC Subunit of H5N1 Viral RNA Polymerase
Source: PLoS One. 2012 Aug 24;7(8):e35234. doi: 10.1371/journal.pone.0035234 (PMC3427309; doi:10.1371/journal.pone.0035234)
Supplement: Table S1 — Structure of candidate compounds. (DOC) [file pone.0035234.s031.doc]

**Table S1. Structure of candidate compounds.**

| 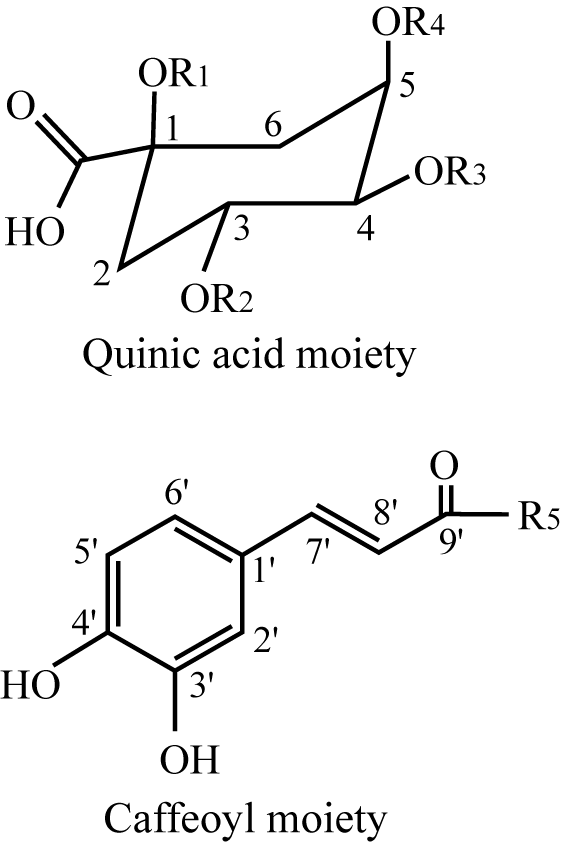 | Candidates | Compounds | R_1_^[a]^ | R_2_ | R_3_ | R_4_ | R_5_^[b]^ |
| --- | --- | --- | --- | --- | --- | --- | --- |
|  | **a** | 3,4-dicaffeoylquinic acid | H | Caffeoyl | Caffeoyl | H | - |
|  | **b** | 1,5-dicaffeoylquinic acid | Caffeoyl | H | H | Caffeoyl | - |
|  | **c** | 4,5-dicaffeoylquinic acid | H | H | Caffeoyl | Caffeoyl | - |
|  | **d** | 3,5-dicaffeoylquinic acid | H | Caffeoyl | H | Caffeoyl | - |
|  | **e** | 1,3-dicaffeoylquinic acid | Caffeoyl | Caffeoyl | H | H | - |
|  | **f** | 5-caffeoylquinic acid | H | H | H | Caffeoyl | - |
|  | **g** | 4-caffeoylquinic acid | H | H | Caffeoyl | H | - |
|  | **h** | quinic acid | H | H | H | H | - |
|  | **i** | caffeic acid | - | - | - | - | OH |

[a] compounds **a**-**h** are based on quinic acid moiety; [b] compound **i** is based on caffeoyl moiety.
