# Supplementary material for: Screen Anti-influenza Lead Compounds That Target the PAC Subunit of H5N1 Viral RNA Polymerase
Source: PLoS One. 2012 Aug 24;7(8):e35234. doi: 10.1371/journal.pone.0035234 (PMC3427309; doi:10.1371/journal.pone.0035234)
Supplement: Table S2 — Binding affinities of chlorogenic acid to different active sites of PAC evaluated by virtual docking. (DOC) [file pone.0035234.s032.doc]

**Table S2. Binding affinities of chlorogenic acid to different active sites of PA_C_ evaluated by virtual docking.**

| Candidates | Compounds | p*K*_d_ | | | | |
| --- | --- | --- | --- | --- | --- | --- |
|  |  | Site 1 | Site 2 | Site 3 | Site 4 | Site 5 |
| **a** | 3,4-dicaffeoylquinic acid | 6.73 | 6.93 | 6.81 | 6.95 | 6.34 |
| **b** | 1,5-dicaffeoylquinic acid | 6.38 | 6.53 | 6.76 | 6.67 | 6.71 |
| **c** | 4,5-dicaffeoylquinic acid | 6.19 | 7.53 | 6.89 | 7.32 | 6.17 |
| **d** | 3,5-dicaffeoylquinic acid | 6.32 | 6.49 | 7.22 | 6.78 | 6.01 |
| **e** | 1,3-dicaffeoylquinic acid | 6.59 | 6.67 | 6.59 | 6.95 | 6.51 |
| **f** | 5-caffeoylquinic acid | 6.01 | 6.56 | 6.36 | 6.15 | 6.1 |
| **g** | 4-caffeoylquinic acid | 6.01 | 6.34 | 5.9 | 5.77 | 5.41 |
| **h** | quinic acid | 5.27 | 5.05 | 5.08 | 4.47 | 4.46 |
| **i** | caffeic acid | 4.77 | 4.8 | 5.53 | 4.88 | 4.45 |
